# A structurally preserved allosteric site in the MIF superfamily affects enzymatic activity and CD74 activation in D-dopachrome tautomerase

**DOI:** 10.1016/j.jbc.2021.101061

**Published:** 2021-08-09

**Authors:** Emily Chen, Krystle Reiss, Dilip Shah, Ramu Manjula, Brandon Allen, Eva L. Murphy, James W. Murphy, Victor S. Batista, Vineet Bhandari, Elias J. Lolis, George P. Lisi

**Affiliations:** 1 Department of Molecular Biology, Cell Biology, & Biochemistry, Brown University, Providence, Rhode Island, USA; 2 Department of Chemistry, Yale University, New Haven, Connecticut, USA; 3 Section of Neonatology, Department of Pediatrics, Cooper University Hospital, Camden, New Jersey, USA; 4 Department of Pharmacology, Yale University School of Medicine, New Haven, Connecticut, USA

**Keywords:** MIF, allosteric regulation, cytokine, CD74, tautomerase, 4-HPP, 4-hydroxyphenyl pyruvate, BAL, bronchoalveolar lavage, CD74, cluster of differentiation 74, DDT, D-dopachrome tautomerase, HPP, hydroxyphenyl pyruvate, MIF, macrophage migration inhibitory factor, TROSY, transverse relaxation–optimized spectroscopy

## Abstract

The macrophage migration inhibitory factor (MIF) family of cytokines contains multiple ligand-binding sites and mediates immunomodulatory processes through an undefined mechanism(s). Previously, we reported a dynamic relay connecting the MIF catalytic site to an allosteric site at its solvent channel. Despite structural and functional similarity, the MIF homolog D-dopachrome tautomerase (also called MIF-2) has low sequence identity (35%), prompting the question of whether this dynamic regulatory network is conserved. Here, we establish the structural basis of an allosteric site in MIF-2, showing with solution NMR that dynamic communication is preserved in MIF-2 despite differences in the primary sequence. X-ray crystallography and NMR detail the structural consequences of perturbing residues in this pathway, which include conformational changes surrounding the allosteric site, despite global preservation of the MIF-2 fold. Molecular simulations reveal MIF-2 to contain a comparable hydrogen bond network to that of MIF, which was previously hypothesized to influence catalytic activity by modulating the strength of allosteric coupling. Disruption of the allosteric relay by mutagenesis also attenuates MIF-2 enzymatic activity *in vitro* and the activation of the cluster of differentiation 74 receptor *in vivo*, highlighting a conserved point of control for nonoverlapping functions in the MIF superfamily.

The human macrophage migration inhibitory factor (MIF) superfamily, comprised of MIF and D-dopachrome tautomerase (DDT, also called MIF-2), is broadly and constitutively expressed with involvement in proinflammatory and host antimicrobial responses (1, 2, 3). Although they are critical immunoregulators, elevated levels of MIF family proteins are implicated in inflammatory diseases such as asthma, acute respiratory distress syndrome (ARDS), and arthritis (4, 5, 6). Antibody neutralization of MIF also decreases inflammatory symptoms in animal models (7, 8). However, the therapeutic potential of targeting MIF proteins is currently obstructed by a lack of molecular level detail about their structures and dynamics, as well as how these properties impact disease-state function.

MIF, first discovered and named for its ability to arrest macrophage migration (9), has a variety of functions dependent upon its localization to the nucleus, cytosol, or extracellular space (10, 11, 12). MIF and MIF-2 are known to overlap in function, including in the transcriptional activation of proinflammatory factors after binding to the type-II receptor cluster of differentiation 74 (CD74), the recruitment of signaling subunit CD44 to the MIF–CD74 complex, activation of extracellular signal-regulated kinases 1/2, and the recruitment of leukocytes (12, 13, 14, 15). These conserved functions are likely a reflection of MIF-2 being the only gene with marked homology to MIF, sharing similar gene and protein structure (16, 17). An evolutionarily conserved proline at the N terminus acts as the catalytic base for a tautomerization reaction of unknown biological significance in both proteins (18). This active site has been the target of many MIF inhibitors, yet it has also been demonstrated that certain small molecules can selectively inhibit MIF or MIF-2 (19). Importantly, there is a connection between the enzymatic site and CD74 binding and activation, shown by MIF mutants (20) and molecular dynamics (MD) simulations (21). Whether a similar connection between the enzymatic and CD74 sites exists in MIF-2 is unknown, but it is important to highlight that predicted models of MIF–CD74 and MIF-2–CD74 have similarities (and differences) in regions that interact with CD74 (22).

Our recent NMR studies and computational modeling of MIF structure and dynamics revealed that the hydroxyphenyl pyruvate (HPP) tautomerase activity of MIF was allosterically regulated. We revealed a strong correlation between MIF residues Pro1 and Tyr99 by MD simulations, showed partial attenuation of tautomerase activity, and identified an allosteric pathway with a series of mutations (23). In another study, Tyr99 was an allosteric regulator for CD74-binding/signaling residues through a different set of amino acids (21). Although this pathway was not as well defined, it appears to branch from Tyr99 and continue along the surface-exposed C terminus of MIF. With the exception of Pro1, the amino acids involved in MIF allostery are not conserved at the same positions in MIF-2, although globally, their homotrimeric structures are nearly identical (Fig. 1*A*). We therefore wondered whether MIF-2 can also support allostery based on structural, but not sequential, homology to MIF and if water networks existed to support enzymatic activity, as in MIF (23).

**Figure 1:**
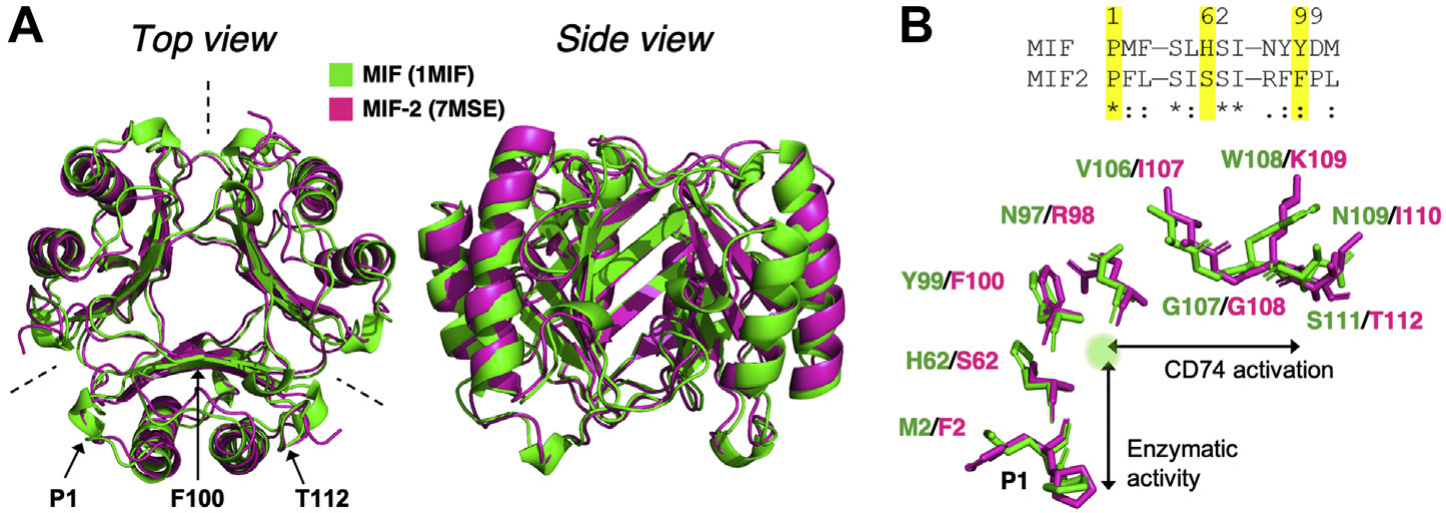
**MIF proteins are conserved in tertiary structure, not in sequence.***A*, X-ray crystal structure overlay of MIF (*green*, PDB ID: 1MIF) and MIF-2 (*pink*, PDB ID: 7MSE). *Dashed lines* indicate the monomer–monomer interfaces, also centered in the side view. The catalytic Pro1, allosteric Phe100, and C-terminal Thr112 residues are indicated for reference. *B*, sequence alignment of key amino acids in the MIF allosteric pathway and side-chain orientation of residues in the pathway originating adjacent to the monomer–monomer interface and traversing the hydrophobic solvent channel (Tyr99/Phe100 allosteric site, *green circle*) and CD74-binding site. *Asterisks* indicate conserved residues, while *dots* indicate similar residues. CD74, cluster of differentiation 74; MIF, macrophage migration inhibitory factor.

We investigated this possibility by introducing mutations in MIF-2 along its hypothesized enzymatic allosteric pathway (Fig. 1*B*). We used NMR spectroscopy, X-ray crystallography, MD simulations, and *in vitro* and *in vivo* functional assays to investigate whether MIF-2 accommodates allostery in a similar fashion to MIF despite a different amino acid sequence. We illuminate a comparable network in MIF-2, where perturbation of these residues altered its local structure, conformational dynamics, and functional responses. Interestingly, we also found that the residues connecting this central allosteric site to the enzymatic site also affected CD74 activation *in vivo*.

## Results

### MIF and MIF-2 have nearly identical structure and function, but different primary sequences

MIF and MIF-2 are highly similar in structure, with monomeric units of 114 and 117 amino acids, respectively, that form homotrimers. Superimposition of the monomeric α-carbons (RMSD < 1 Å) (17) shows a conserved tertiary fold with a hydrophobic core and tautomerase active site at the monomer–monomer interfaces of the quaternary structure (Fig. 1*A*). Interestingly, the MIF and MIF-2 primary sequences are only 35% identical and the amino acids occupying the positions responsible for allosteric regulation of MIF catalysis and CD74 activation are different in MIF-2 (Fig. S1*A*). With the exception of Pro1 and a few other residues (Lys32, Ile64), the enzymatic sites of MIF-2 and MIF are electropositive and hydrophobic, respectively, yet they both catalyze the enol-keto tautomerization of an HPP “pseudosubstrate” (24). Although the physiological relevance of MIF enzymatic activity is unknown, it is surprising that the residues for the enzymatic site and CD74 activation site, a crucial component of immunoregulation (25), are not conserved. Some of the other nonconserved residues may account for activation of the chemokine receptors CXCR2 and CXCR4, signaling activities in the cytosol, and a nuclease activity that mediates cell death, all of which appear to be exclusive to MIF (10, 11, 15).

To further characterize the similarities and differences between these two proteins, we examined whether an allosteric site found in MIF also exists in MIF-2, connected to Pro1 through residues we identified in the solvent channel by mutagenesis (23). The allosteric site of MIF is Tyr99, which gates the solvent channel formed by the trimeric structure and is evolutionarily conserved among MIF proteins in virtually all species. The same site in MIF-2 is Phe100 and is also evolutionarily conserved among all MIF-2 proteins, suggesting this site plays an important role in both proteins (Fig. 1*B* and Fig. S1). Crystal structures of the wt proteins show that the side chains of wt-MIF and wt-MIF-2 are similarly oriented, suggesting that the intermolecular forces stabilizing both relays between the enzymatic and allosteric sites are consistent (Fig. 1*B*). In addition, the Pro1 deep within the active site is coupled to CD74 binding and signaling distal to the MIF enzymatic site. Using these structural cues and functional overlap, we speculated that MIF-2 uses a similar mechanism to couple its N-terminal catalytic site to an allosteric site. Because a correlation between the enzymatic site and CD74 binding/activation was previously shown for MIF (20), we also hypothesized that the allosteric network between the solvent channel and Pro1 affected CD74 function in MIF-2.

### Disruption of the allosteric site in MIF-2 diminishes enzymatic activity

**Figure 2:**
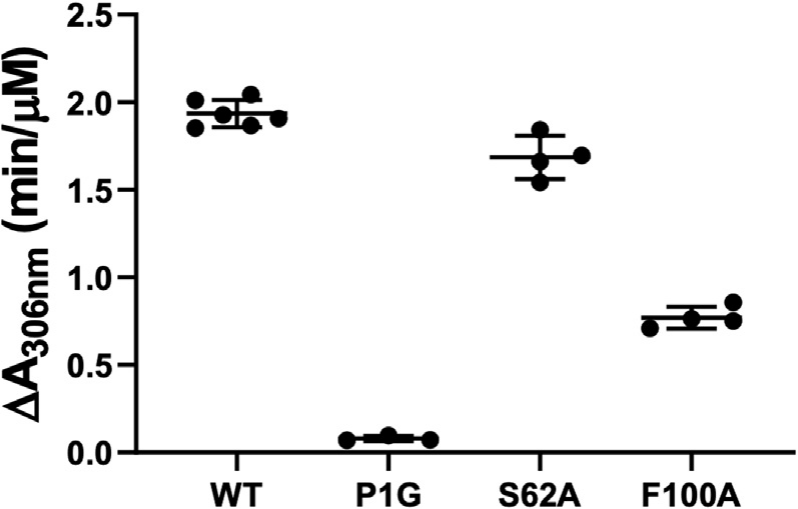
**Mutations in MIF-2 allosteric network attenuate enzymatic activity.** Tautomerase assay monitoring the enol–borate complex of HPP (λmax = 306 nm) produced by MIF-2. n = 3 to 4 in each case, and data scatter displays error between assays. HPP, hydroxyphenyl pyruvate; MIF-2, macrophage migration inhibitory factor 2.

We mutated the key residues in MIF-2 (Pro1, Ser62, Phe100) that occupy the same positions as those in the previously studied MIF allosteric pathway (Pro1, His62, and Tyr99) to probe the existence and potential conservation of allostery in the MIF superfamily (Fig. 1*B* and Fig. S2). The variants P1G, S62A, and F100A were used in MIF-2 tautomerase assays. These variants maintained similar secondary structures and thermal stabilities to wt-MIF-2, suggesting that the mutations did not cause any significant energetic changes to its structure (Fig. S3). Thus, any effect of these mutations on catalytic activity would arise from changes in local structure or dynamics rather than from disruption of the global fold of MIF-2. Mutation of Pro1 abolished activity, supporting the critical role of the N-terminal proline (18) in the HPP enol-keto tautomerase reaction for MIF-2 (Fig. 2). In agreement with MIF enzyme kinetics (23), the activity was decreased in the S62A and F100A variants by 13% and 60% (*versus* wt-MIF-2), respectively. The substantial change in F100A enzymatic activity despite ∼9 Å between the mutation and catalytic sites implies long-range communication within the MIF-2 structure.

### Crystal structures of MIF-2 variants highlight an altered local conformation at the solvent channel

**Figure 3:**
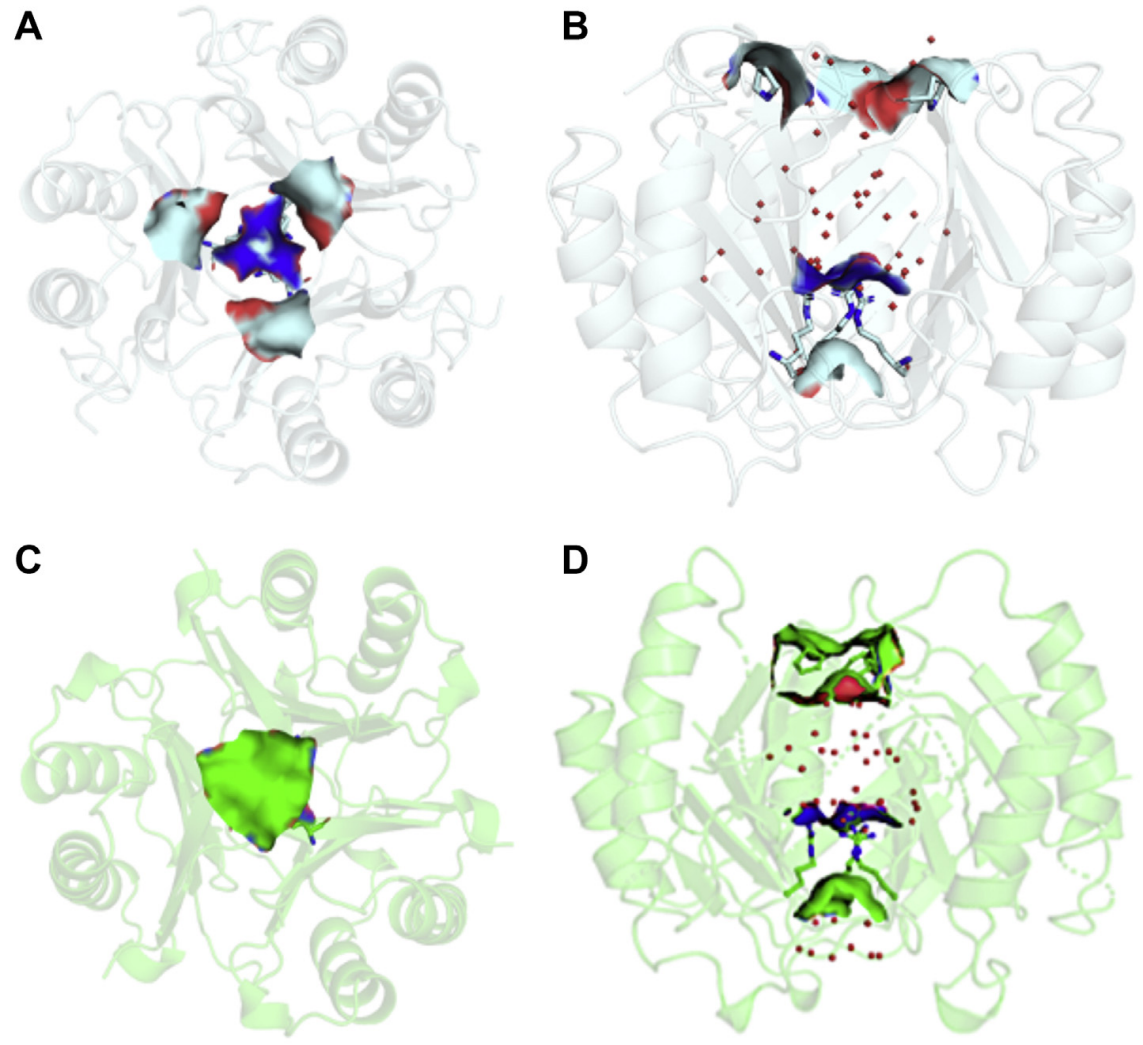
**The solvent cavity of wt-MIF-2 (PDB ID:**7MSE**) and the F100A variant (PDB ID:**7MRV**).***A*, surface area of wt-MIF-2 showing Phe100 (*cyan*) defining the opening of the cavity and Arg42 (*dark blue*) at the *bottom*. *B*, a 90° rotation of panel *A* showing the Arg42 side chains that plug the potential solvent channel. *C*, Pro101 (*green surface*) moves to the center of the cavity and blocks the view of Arg42 below when the F100A variant is oriented similarly to wt-MIF-2 in panel *A*. *D*, a 90° rotation relative to panel *C*, showing the surface area of Arg42 and Pro101 with trapped water molecules (*reddots*). MIF-2, macrophage migration inhibitory factor 2.

We characterized the three-dimensional structures of MIF-2 variants to determine whether there were any changes in conformation or within water networks of the solvent channel as seen in MIF and related to enzymatic activity (crystallographic data are shown in Table S1). MIF-2 variants were compared with the wt-MIF-2 structure (PDB ID: 7MSE), which we solved to higher resolution (1.27 Å) to resolve water networks with more certainty than the previously published structure (PDB ID: 1DPT) (17). No significant deviations from the previous structure were noted. We determined the RMSD for the variants *versus* wt-MIF-2 and also compared wt-MIF-2 structure with wt-MIF. A major difference between MIF-2 and MIF is the replacement of the solvent channel of MIF with a large solvent cavity in MIF-2 (Fig. 3). This new observation provides a modest level of structural differentiation within the MIF superfamily, based on the identity of the gating residues. In MIF, the top (active site face) and bottom of the channel are gated by Tyr99 and Val42/Gln45 with diameters of ∼3.5 Å and ∼8.0 Å at each opening, respectively. In MIF-2, the lower gating residue is instead Arg42, and its longer side chains from the three subunits prevent a solvent channel from fully forming.

Crystal structures of MIF variants and substrate- or inhibitor-bound complexes typically display very few differences, and consistent with this trend, the P1G and S62A variants have RMSD values of 0.30 Å and 0.32 Å with the wt-MIF-2 trimer, respectively (Fig. S4*A*). The P1G MIF-2 variant has no catalytic activity because of the dynamics of the glycine and the altered p*K*a preventing it from functioning as a catalytic base, which is also observed for P1G MIF (26). This conclusion is supported by the F100A and S62A variants, which should have the same proline p*K*a as wt-MIF-2 and possess catalytic activity. We were surprised to find the initiating Met of the P1G MIF-2 variant, as it is cleaved by *Escherichia coli* methionine aminopeptidase in the P1G MIF protein. Our goal with the P1G variant was to abolish activity and probe the catalytic site's effect on Phe100, and the unexcepted variant with the initiating Met still serves that role. The N-terminal nitrogen of F100A and S62A differs from that of wt-MIF-2 by 1.7 Å and 0.8 Å, respectively. These differences are influenced by tartrate in the wt-MIF-2 active site with two hydrogen bonds from the Pro1 nitrogen to the tartrate carboxylate (2.9 Å) and carbonyl (2.7 Å) oxygens that stabilize its position. wt-MIF-2 is the only protein in this study that has tartrate in its crystallization conditions.

The MIF-2 S62A variant was made to test this serine as a transmission conduit between Phe100 and Pro1 in MIF-2, equivalent to His62 bridging Tyr99 and Pro1 in MIF. The loss in enzymatic activity of MIF-2 S62A (13%) is qualitatively similar to that of MIF H62A (25%), relative to the activities of their respective wt proteins. Ser62 and His62 are not part of the active sites, with side chains that point into the solvent cavity or channel, respectively, and are not expected to cause any enzymatic activity loss unless each residue is involved in the transmission pathway between the allosteric and active sites. It must be noted that Ser62 interacts with Phe2, adjacent to Pro1, meaning the impact of the S62A mutation on catalysis could in part be due to loss of communication between Ser62 and Phe2 that propagates to Pro1. The same scenario is also true for MIF. However, the crystal structure of S62A MIF-2 shows no evidence of alteration to the Phe2 side chain relative to that of wt-MIF-2 (Fig. S5), suggesting this factor may not have a large influence.

Surprisingly, the F100A variant has a higher RMSD (1.245 Å) with wt-MIF-2, displaying substantial conformational changes in the β-strand housing the F100A mutation, the C-terminal region, and the loop 63 to 69 connecting the third β-strand to the second α-helix (Fig. S4*B*). These changes were not observed in the Y99A MIF mutant, but in MIF-2, this mutation induces the adjacent Pro101 from each subunit to form a “lid” over the solvent cavity to separate external and internal water molecules (Fig. 3, *C* and *D*). To understand the loss of ∼60% of enzymatic activity in F100A relative to wt-MIF-2, the superimposed wt-MIF-2 and F100A structures were examined using tartrate (from crystallization conditions of wt-MIF-2) in the electropositive active site as a guide. Seven wt-MIF-2 residues with atoms within 4 Å of the tartrate were compared with the same residues of F100A (Fig. S4*C*). There are significant differences in the F100A variant including a conformational change in the active site residue Ile64 and increased dynamics of the active site Arg36 and C-terminal Met114 side chains (dynamics are interpreted from the absence of electron density). Interestingly, the changes caused by the F100A mutation are reminiscent of a cysteine mutation in MIF designed to create an intersubunit disulfide, which caused conformational changes in the loop linking the third β-strand to the second α-helix and the C-terminal region (27), implying a propensity for dynamics at these sites. This Cys mutation also induced a “trimer of MIF trimers” oligomerization as determined by NMR and crystallography and decreased MIF enzymatic activity to a much greater extent relative to the wt protein (k_cat_/K_m_ of 6%) (23). We conclude that the decrease in enzymatic activity for F100A MIF-2 may be due to changes in the active site architecture, but to probe these changes further, we examined its solvent cavity water network, which we previously hypothesized to play a role in MIF enzymatic activity (23).

### Experimental and computational water molecule analysis shows subtle differences in MIF-2 variants

**Figure 4:**
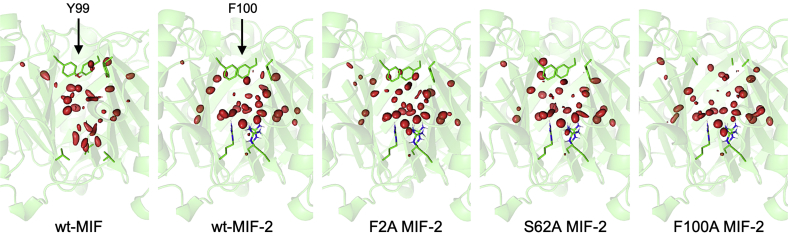
**Models of 150-ns MD simulations examining water molecule occupancy in wt-MIF, wt-MIF-2, and the MIF-2 variants F2A, S62A, and F100A.** Water molecules within the solvent channel, as well hydrogen bond donor–acceptor distances between gating residues Y99–V42 (MIF) and F100–Arg42 (MIF-2) at the *top* and *bottom* of the channel, were used to determine the number of water molecules in the cavity of each MIF/MIF-2 model. MIF, macrophage migration inhibitory factor; MIF-2, macrophage migration inhibitory factor 2.

In our prior study of MIF, we speculated that solvent channel water molecules and the size of the opening to the channel could mediate catalysis because of an observation that increasingly dense and ordered water networks and smaller gates of the solvent channel pore correlated with greater catalytic rates (23). Here, two parameters were used to examine the same possibility in MIF-2 and compare our findings to MIF: water molecule occupancy and hydrogen bonding around the channel. We first examined the MIF-2 solvent cavity for comparison to that of MIF by performing MD simulations on P1G, F2A, S62A, and F100A MIF-2 variants, as well as F2M, S62H, and F100Y variants that substitute the MIF residues into MIF-2. Calculations noted a 1.3 Å expansion of the upper solvent channel in wt-MIF-2 as compared with wt-MIF, based on the distance between the average Cα positions of Tyr99/Phe100 (Fig. S6). These dynamics are not captured by X-ray structures of either protein. Mutations at MIF-2 allosteric nodes (S62H, S62A, F100Y) all retain this partial expansion. F2M and F2A variants only expand by 0.9 Å and 0.8 Å, respectively, indicating that a phenylalanine at position 2 is at least partially responsible for pushing the channel outward in MIF-2 (this residue is Met2 in MIF). In addition, the expansion in the F100A variant is also only 0.9 Å due to the loss of the large side chain near the mouth of the cavity and movement of Pro101 into this space, a conformational change not observed in Tyr99 MIF variants. Because it is primarily the addition of a large side chain at Phe2 that pushes the channel outward, the solvent cavity of wt-MIF-2 does not contain any more waters than wt-MIF (41 *versus* 42) despite the expansion in the upper cavity. This is also likely because the bottom of the MIF-2 channel is largely blocked by Arg42, causing this cavity to appear truncated relative to MIF, which instead has a valine at the same position. Although the number of waters is similar, how these waters fill the solvent cavity is remarkably different (Fig. 4), consistent with crystallographic data. Several waters are instead able to fit in the space between the MIF-2 monomers because of the change of Leu58 in MIF to a more flexible and polar Gln58 in MIF-2.

Another interesting feature is the dense region of waters in a horizontal layer just above the Arg42 side chain of MIF-2. This is due to an increase in charged, hydrophilic residues in this area of MIF-2 (Arg42, Glu4), while MIF has the hydrophobic Leu and Ile residues at these positions. All simulated MIF-2 variants show very similar cavity structures to that of wt-MIF-2, which contrasts MIF variants that underwent significant restructuring of the solvent network (23). This may be explained by the fact that the MIF channel is much more open to external solvent (Fig. 4). Overall, calculations highlight how MIF and MIF-2 maintain identical volumes in their solvent cavities, despite being filled quite differently. Because prior MD studies revealed these waters to facilitate intermonomer crosstalk (21, 23), we speculate that differences in the amino acid sequences of the MIF and MIF-2 proteins may drive subtle changes to the solvent arrangement to modulate the exact impact of each residue in functional assays.

We next assessed the crystallographic water networks for wt-MIF-2 and three variants and found wt-MIF-2 to have the densest network of water molecules (Fig. 3). P1G also has an extensive water network, but whatever effect this might have on catalytic activity is abolished by the removal of Pro1. However, the catalytically active S62A shows a water network slightly less dense than wt-MIF-2/P1G, correlating with its modestly attenuated activity. There is an even slightly smaller network of water molecules trapped between Pro101 and Arg42 in F100A MIF-2 (Fig. 3*D*), which has a *k*_cat_/*K*_m_ ∼40% of wt-MIF-2. It must be noted that changes in the F100A water network are partly due to the truncation of the solvent cavity from multiple conformational changes. Thus, we can only associate strong water networks in MIF-2 with the catalytically active wt-MIF-2 and S62A, while the weaker activity of F100A shows a slightly diminished solvent network. These observations, and differences in the architecture of the MIF and MIF-2 solvent channels, make it difficult to conclusively identify patterns in the solvent arrangement that correlate with MIF-2 catalysis. Our laboratories are exploring orthogonal methods to determine the mechanistic impact of this channel within the MIF superfamily. The fact that MIF and MIF-2 catalyze the same HPP substrate, but with hydrophobic and electropositive active sites, respectively, could suggest the role of the solvent channel is slightly different in these systems.

### Critical hydrogen bonds of MIF are preserved in MIF-2

Previously, Tyr99 in MIF was shown to be connected to the Pro1 active site through hydrogen bonds between three neighboring beta strands (23). In MIF-2, these donor–acceptor pairs are Phe2–Ser62, Ser62–Phe2, Ser63–Phe100, and Phe100–Ile61. To determine if changes in hydrogen bonding along the MIF-2 solvent cavity could reveal differences in the MIF and MIF-2 allosteric pathways, we again turned to MD simulations. Comparing MIF to MIF-2, we observe a decrease from 0.83 hydrogen bonds per frame to 0.71 hydrogen bonds per frame (MIF/MIF-2) from His62–Met2 or Ser62–Phe2 (Fig. S7). However, when these positions (2 and 62) swap the donor–acceptor, MIF-2 increases the interaction from 0.71 to 0.80. This means that although the nature of the His62–Met2 and Ser62–Phe2 interaction changes, there is no net quantitative change in these two residues between MIF and MIF-2. Conversely, the hydrogen bond between His62–Tyr99 and Ser63–Phe100 is practically erased in MIF-2 from 0.38 bonds/frame to 0.05 bonds/frame. This hydrogen bond is partially recovered (0.19 bonds/frame) in a MIF-2 S62H mutant, suggesting it is the loss of histidine that destroys this interaction, as the imidazole ring is able to stack with Tyr99 or Phe100. The fourth hydrogen bond, Tyr99–Leu61 or Phe100–Ile61, is unchanged (0.75–0.73) between MIF and MIF-2, which is unsurprising, considering that both have very similar residues at these positions. Taken together, the greatest difference in the pathway between the Pro1 active site and Tyr99 or Phe100 is in a hydrogen bond that is not particularly strong in either enzyme (His62–Tyr99 or Ser63–Phe100), implying that the allosteric pathway is effectively unchanged from a hydrogen-bonding standpoint.

### NMR chemical shift correlations reveal structural and dynamic perturbations along the allosteric pathway

**Figure 5:**
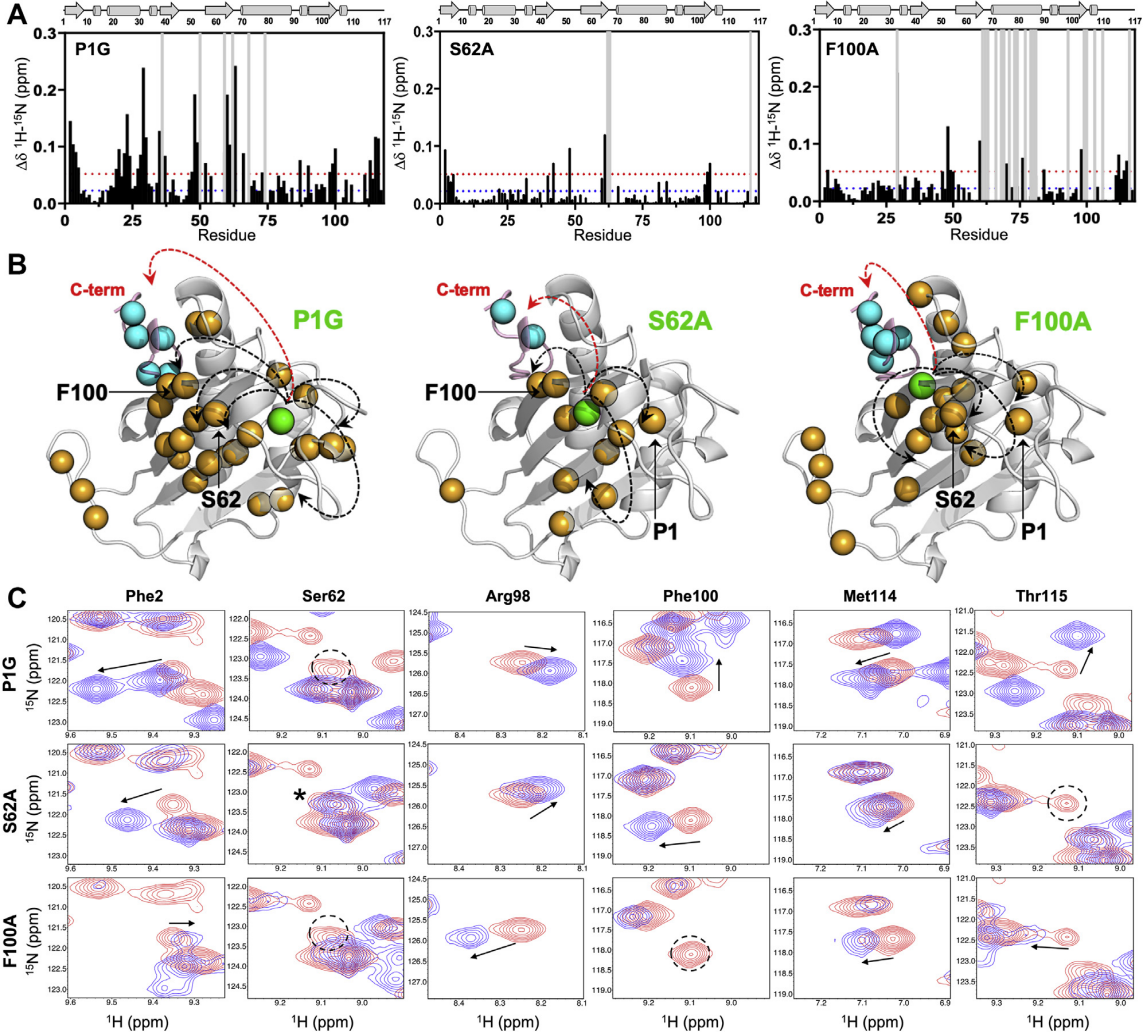
**Global and allosteric perturbations in the MIF-2 structure detected by NMR.***A*, combined ^1^H^15^N chemical shift perturbations (*black bars*) in MIF-2 caused by mutations. *Blue* and *red dashed lines* represent the 10% trimmed mean and 1.5σ above the mean, respectively. *Gray bars* denote regions of NMR line broadening. *B*, NMR chemical shifts (+1.5σ) and line broadening are mapped onto the MIF-2 structure (*orange spheres*). The site of mutation is shown in *green*, and the C terminus of the adjacent MIF-2 monomer, believed to be the site of CD74 activation, is shown in *pink* with *blue spheres*. *Arrows* indicate short- and long-range correlations relative to the site of mutation. *C*, ^1^H^15^N NMR spectral overlays of wt-MIF-2 (*red*) and its variants (*blue*). The mutation corresponding to each row of data is listed at the *left*. Perturbations from the wt to mutant are highlighted with a *black arrow* (shift) or *dashed circle* (broadening). ^∗^Val39 and Glu4 shift strongly to overlap with the vacant Ser62 resonance in the S62A variant. Each mutation affects residues believed to be coupled within the allosteric pathway (Phe2, adjacent to Pro1; Ser62; Phe100) as well as C-terminal residues in proximity of the CD74 site based on studies with MIF (Arg98; Met114; Thr115). Full NMR spectral overlays are provided in Fig. S8. CD74, cluster of differentiation 74; MIF-2, macrophage migration inhibitory factor 2.

Solution NMR was used to determine whether potential allosteric pathway residues were critical to intramolecular communication of MIF-2. Chemical shift profiles from ^1^H^15^N transverse relaxation–optimized spectroscopy (TROSY)–heteronuclear single-quantum coherence spectra show varying degrees of perturbation and line broadening, a signature of heightened dynamics, across these variants (Fig. 5*A* and Fig. S8). These effects are propagated to distant regions of the protein, well beyond the site of mutation and consistent with prior observations for MIF. Proline 1 mutation has a large influence on the MIF-2 structure in solution, whereas the F100A mutant had more modest chemical shift perturbations by comparison. However, F100A displays more line broadening, particularly in residues 60 to 65 that were noted to undergo a conformational change in the F100A crystal structure. Beyond this region, the F100A variant had the greatest overall attenuation of NMR intensity across the MIF-2 protein (Fig. S9). The S62A variant displayed the weakest structural and dynamic perturbations (Fig. 5*A*). While a Pro1 mutation was expected to severely abrogate enzymatic function because of its role as a catalytic base, the spatially distinct F100A variant shows a large decrease in enzymatic activity, coupled to substantial NMR line broadening, while S62A displays only modest solution perturbations, has a lesser effect on NMR peak intensities, and is closer to wt-like activity (Fig. 2). A further analysis of correlated NMR chemical shifts confirmed that mutations at these residues strongly perturb others in/adjacent to the pathway (*i.e.*, “reciprocity”), as seen in NMR spectral overlays of wt-MIF-2 *versus* its variants (Fig. 5*B*). These data are consistent with prior conclusions for MIF, which displayed chemical shift correlations among all residues in its allosteric pathway and an inverse correlation between structural dynamics and catalytic activity (23).

### NMR spin relaxation experiments highlight long-range dynamic perturbations

**Figure 6:**
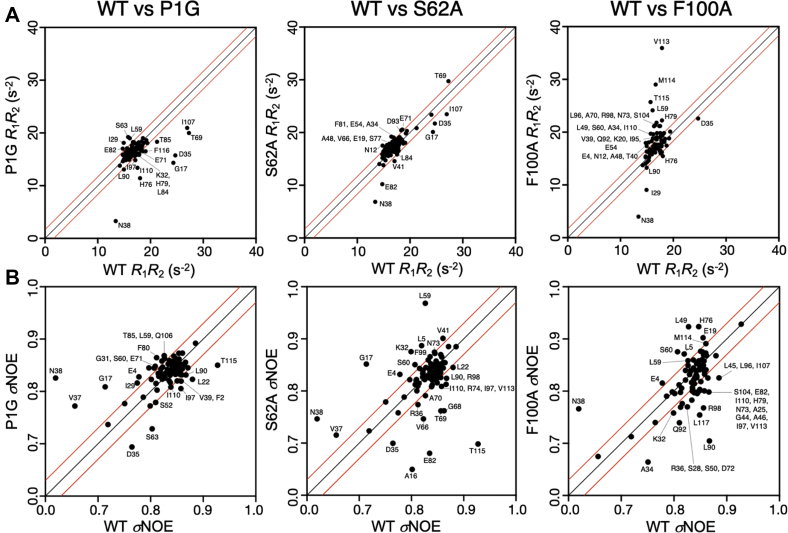
**Relaxation parameters for wt-MIF-2 and allosteric pathway variants.***A*, *R*_1_*R*_2_ correlations between wt and P1G, S62A, and F100A MIF-2. Spearman’s Rho coefficients for entire datasets are 0.45 (P1G), 0.76 (S62A), and 0.53 (F100A). *B*, ^1^H-[^15^N] NOE correlation between the same samples. Spearman’s Rho coefficients for entire data sets are 0.46 (P1G), 0.38 (S62A), and 0.48 (F100A). In panels *A* and *B*, points that diverge from the linear regression fit indicate differences in NMR-derived relaxation parameters at specific residues in these samples. *Black lines* indicate perfect correlation between experimental values (*i.e.*, x = y), and *red lines* denote 1.5σ boundaries from the 10% trimmed mean of all relaxation data. MIF-2, macrophage migration inhibitory factor 2.

NMR was also used to probe the dynamics of wt-MIF-2 and its variants through *T*_1_, *T*_2,_ and ^1^H-[^15^N] NOE relaxation experiments to better understand the consequences of perturbing this allosteric pathway. The resulting *R*_1_ and *R*_2_ relaxation rates (Tables S2–S5 and Fig. S10) are displayed in Figure 6*A* as the *R*_1_*R*_2_ product to suppress contributions from anisotropic molecular tumbling (28). The *R*_1_*R*_2_ values were plotted on correlation diagrams between wt-MIF-2 and its variants to determine the extent to which mutations affected MIF-2 dynamics (Fig. 6). In this analysis, residues with relaxation parameters that significantly deviate from a linear correlation (*i.e.*, outside 1.5σ of the 10% trimmed mean) are suggested to be most strongly affected by mutations along the allosteric pathway. These residues are mapped onto the MIF-2 trimeric and monomeric structures in Fig. S11. Sites of line broadening in chemical shift plots of P1G MIF-2 do not overlap that strongly with those showing altered dynamic parameters in relaxation experiments, suggesting chemical shift modulations are averaged out on the timescale of the experiment. However, subtle elevation in the dynamics of residues 20 to 30 distal to the monomer–monomer interface and decreases in residues 60 to 75 surrounding the allosteric pathway along the solvent cavity are observed in P1G MIF-2 (Fig. S10). Consistent with its modest effect on MIF-2 chemical shifts and enzymatic activity, S62A displays *R*_1_*R*_2_ parameters that deviate least from wt-MIF-2. The P1G and F100A variants show similar levels of dynamic perturbation (Fig. 6*A*); however, the effects of F100A are strongest at the C terminus, most notably residues Ile107 and Ile110, which have been purported to be involved in CD74 activation (Fig. S10). More subtle alterations in *R*_1_*R*_2_ parameters are observed in Ile63 and Met114, identified in crystal structures to have conformational plasticity. Elevated *R*_2_ values surround regions of line broadening observed in NMR spectra of S62A and F100A MIF-2 (residues 60–65, particularly), suggesting a change in *R*_ex_ that is also consistent with nearly all deviations from wt-like dynamics in these variants being driven by *R*_2_ (*i.e.*, elevated *R*_1_*R*_2_, Fig. S10).

Correlation plots of ^1^H-[^15^N] NOE, reflecting differences in *ps*-*ns* dynamics between wt-MIF-2 and variants, generally look similar across the samples (Fig. 6*B*, Figs. S12 and S13*B*), suggesting that motions probed by the ^1^H-[^15^N] NOE experiment do not differentiate MIF-2 variants as significantly. Locally altered regions of flexibility include the monomer–monomer interface (residues 25–36; Arg36 lacked electron density in the F100A X-ray structure), extended solvent cavity (residues 75–100), and very slight changes at the C terminus. Considering NMR line broadening and spin relaxation data, larger degrees of dynamic change induced by MIF-2 variants are correlated with the loss of enzymatic activity, following a trend P1G >F100A >> S62A. This trend is qualitatively similar to that reported for MIF (23).

### Correlated motions connecting critical regions of MIF-2 are altered by mutations

To quantitate the impact of mutations on intramonomer and intermonomer correlations in MIF-2, we analyzed the final 100 ns of MD simulations on variants that mimic wt-MIF (S62H, F100Y) as well as the experimentally characterized P1G, S62A, and F100A variants. Variants that mimic MIF show an increase in the correlated motions of all monomers throughout the majority of MIF-2 (Fig. S14). This effect can be loosely associated with the higher basal catalytic activity of MIF *versus* MIF-2 but more strongly corroborates dynamic trends measured by NMR in our previous study of MIF. In contrast, heat maps generated from MD simulations demonstrate that correlated motion within MIF-2 is disrupted by the P1G, S62A, and F100A mutations. Indeed, intermonomer crosstalk is attenuated in the β2-4/α3 region (residues 57–63 and 70–85, Fig. S14) and the C-terminal region (residues 98–117), suggesting an allosteric effect is propagated from the mutation site. Specific disruptions of correlated motion between the active and allosteric sites are observed, most notably between residue pairs 2 to 100, 42 to 100, and 62 to 100. Consistent with NMR chemical shifts, correlation matrices show the effect of S62A on these sites to be more modest, which also reflects its near wt levels of catalytic activity.

### MIF-2 variants are impaired in their ability to activate the CD74 receptor *in vivo*

**Figure 7:**
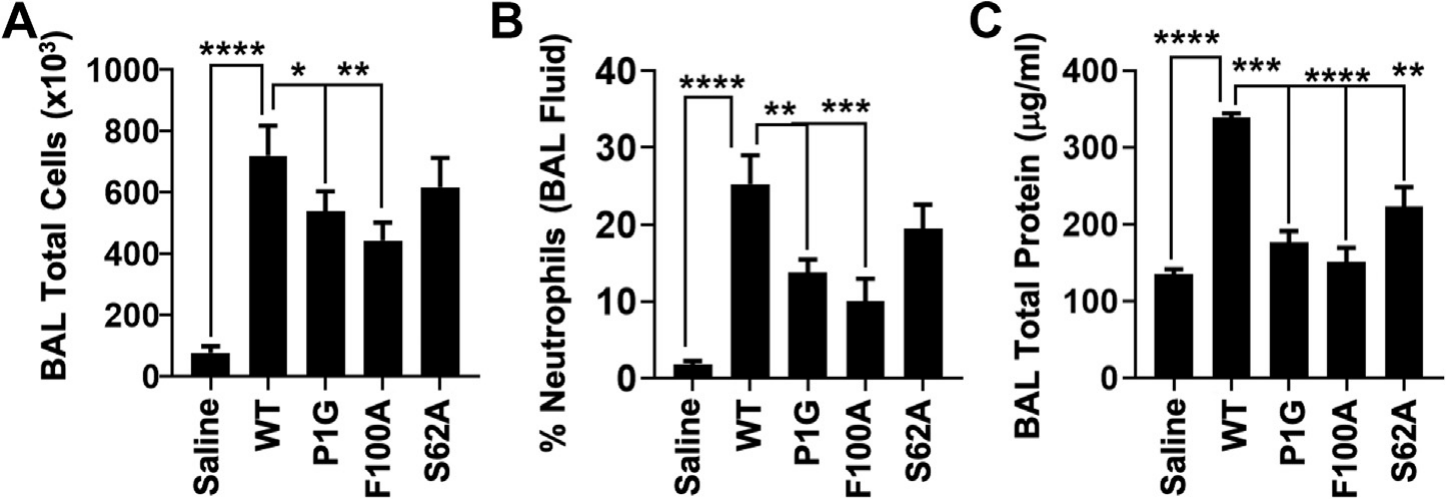
**Allosteric mutations decrease the ability of MIF-2 to generate an inflammatory response *in vivo*.***A*, MIF-2 variants P1G and F100A significantly decreased the total BAL cell counts *in vivo*. *B*, MIF-2 variants P1G and F100A significantly diminished neutrophil recruitment in BAL fluid *in vivo*. *C*, MIF-2 variants P1G, S62A, and F100A led to significantly decreased BAL fluid total protein levels, as a surrogate marker for alveolar–capillary leak/pulmonary edema *in vivo* (n = 4 in each group, ∗*p* < 0.05, ∗∗*p* < 0.01, ∗∗∗*p* ≤ 0.001, and ∗∗∗∗*p* ≤ 0.0001). Data are expressed as the mean ± SEM. BAL, bronchoalveolar lavage; MIF-2, macrophage migration inhibitory factor 2.

The ability of the MIF family of proteins to stimulate neutrophil recruitment into murine lungs has been a well-established marker for the activation of the proinflammatory CD74 receptor *in vivo* (29). Prior work with MIF demonstrated the solvent channel residue Tyr99 was allosterically coupled to the CD74-binding site, as the Y99A mutation abolished neutrophil recruiting activity (21). The allosteric effect of this mutation appeared to be propagated through a different pathway than that affecting Pro1 and enzymatic activity (21). The allosteric pathway governing enzymatic function, which is the subject of our present study and previously included the imidazole side chain of His62 (adjacent to Tyr99) at the solvent channel interface (23), has never been tested for an effect on neutrophil recruiting activity in MIF or MIF-2. In probing the Pro1, Ser62, and Phe100 residues in MIF-2, we evaluated the effect of mutations on neutrophil recruitment into murine lungs and resulting pulmonary edema. We measured the total cell count, percentage of neutrophils, and total protein content (as a surrogate marker of alveolar–capillary leak/pulmonary edema) in the bronchoalveolar lavage (BAL) fluid obtained from mice. The mice were sacrificed after 6 h of administration of a vehicle (saline) only or vehicle + wt-MIF-2 or one of the MIF-2 variants: P1G, S62A, and F100A. wt-MIF2 significantly increased the total BAL fluid cell counts, whereas the MIF-2 variants P1G and F100A lead to a significantly decreased total BAL fluid cell count (Fig. 7*A*). The significant impact of these residues on MIF-2 function is consistent with enzymatic assays that correlate inversely with heightened dynamics in NMR experiments.

The neutrophil influx (Fig. 7*B* and Fig. S15) and BAL fluid protein levels (Fig. 7*C*) were significantly increased in the wt-MIF-2 group (compared with controls) but significantly decreased in groups administered MIF-2 variants. Taken together, these findings indicate that MIF-2 variants (specifically, P1G and F100A) have a functional effect in reducing the inflammatory response in the murine lung. While the inflammatory cellular response was modestly impacted by S62A administration, it did decrease alveolar–capillary leak in the murine model, when compared with wt-MIF2.

## Discussion

We used an integrated approach of X-ray crystallography, MD simulations, NMR, and *in vitro* and *in vivo* functional assays to probe the existence of allostery in MIF-2, the only other human homolog of MIF that shares nearly identical structure despite low sequence conservation. Consistent with our previous work investigating MIF allostery, mutations in analogous MIF-2 residues affected structure, dynamics, and biological function, although to somewhat different levels than that observed in MIF. The human MIF superfamily also has two bacterial homologs, 5-(carboxymethyl)-2-hydroxymuconate isomerase and 4-oxalocrotonate tautomerase (30), and an additional 4-oxalocrotonate tautomerase homolog (31) with similar tertiary folds of dimeric, trimeric, and hexameric quaternary structures, but with 5 to 20% primary sequence homology to MIF and MIF-2. The presence of an allosteric network in these more distant homologs is currently unknown, but they all share a conserved Pro1 and active sites at the monomer–monomer interface. There appears to be an evolutionary pressure in MIF to retain Pro1 with a low p*K*a (26), consistent with an enzymatic reaction, but there has been debate as to whether this is due to Pro1 being a vestigial artifact or a catalytic base for a physiological reaction (20). Another argument is that Pro1 is retained as a portion of a dynamic pathway that imposes structural and functional influence over the MIF superfamily. Our work cannot comment on a physiologically relevant reaction but does provide support for the second claim.

The MIF-2 variants P1G, S62A, and F100A demonstrated that alterations to a hypothesized allosteric pathway disrupt enzymatic function *in vitro* with HPP tautomerase assays. The abolished activity of the proline variant comes with no surprise because of its role in catalyzing the reaction. Both S62A and F100A had decreased activity compared with wt-MIF-2, similar to MIF variants at the same positions, H62A and Y99A (23). In our analysis, the decrease in activity for S62A and F100A in MIF-2 were 13% and 60%, respectively. In MIF, this was previously reported as a decrease of 25% and 38% for H62A and Y99A, respectively. Here, alteration of residue 62 resulted in similar trends of decreased activity, although part of this effect may be due to diminished interaction with residue 2. However, the F100A variant in MIF-2 had a more drastic effect on activity than the MIF Y99A counterpart. This was unexpected because MIF tautomerizes HPP with a specific activity roughly 10-fold higher than MIF-2 (3) and Tyr99 was determined through MD simulations to be a highly influential allosteric node in MIF (21). Before our X-ray structure of F100A MIF-2 showed significant conformational changes, it was unclear why a Phe100 mutation would have a larger effect on the activity in MIF-2. As stated earlier, these unanticipated structural alterations are reminiscent of a single cysteine substitution in MIF that causes conformational rearrangement of the active site and C terminus, but retains attenuated catalytic activity (27), which could partially inform our observations in F100A MIF-2.

NMR supports the connection of these MIF-2 residues through correlated chemical shifts and spin relaxation, where each mutation perturbed other residues in or adjacent to the proposed network. *In vitro* assays demonstrated that the tautomerase active site is sensitive to mutations at Ser62 and Phe100 (Fig. 2); however, it is clear from NMR spectral overlays of wt-MIF-2 and other variants that the dynamic communication of the residues occurs reciprocally. The P1G variant logically perturbed Phe2; however, it also caused broadening at Ser62 and chemical shift perturbations at Phe100 and selected sites near flexible loops and the C-terminus. S62A and F100A variants similarly perturb Pro1 and Phe2. Aside from the mutual perturbations, the variants also propagate structural and dynamic changes to distal regions of MIF-2 (*i.e.*, subunit interfaces), indicated by the extent of chemical shifts and line broadening across the entirety of the protein (Fig. 5), including at regions of conformational change in X-ray structures. This effect is also observed in MD analysis of correlated motion within the MIF-2 monomers. Consistent with our prior studies of MIF, mutations that induce greater flexibility in the enzyme diminish catalytic activity to a greater degree, as seen in F100A *via* line broadening and elevated *R*_1_*R*_2_ in the purported CD74-binding site and subunit interface. In addition, residues previously identified to be critical for CD74 activation along a computationally derived regulatory pathway in MIF (21, 32) were all perturbed structurally or dynamically in MIF-2 (Arg98, Ile107, and Ile110, Figs. S10 and S12*A*). Furthermore, MD simulations of MIF-2 variants revealed correlated motions to be disrupted at the C terminus, suggesting communication between the active, allosteric, and purported CD74-binding sites.

The MIF family of proteins are constitutively expressed, secreted in response to stress to circulate in the body at low concentrations (33), and bind CD74 to promote a range of inflammatory (25), protective, or repair responses (34). Inhibitors of the catalytic site disrupt CD74 activation by blocking surface interactions with the receptor *via* steric hinderance (32). However, the extensive functions of MIF proteins require more stringent regulatory control within the core of its structure. Altogether, our data support the preservation of an allosteric pathway in MIF-2 preserved within its structural fold despite differences in amino acid sequence in the related human MIF proteins. Identification of novel allosteric pathways in MIF and MIF-2 provide an alternative target for drug design and fine-tuning CD74 activation, which can now be explored at the molecular level in future work with MIF and MIF-2.

## Experimental procedures

### Protein expression and purification

wt and mutant MIF-2 proteins were expressed and purified as previously described (17). Specifically, MIF-2 proteins were transformed into *E. coli* BL21-Gold (DE3) cells and grown at 37 °C to an absorbance of ∼0.6 at 600 nm. MIF-2 was induced at a final concentration of 1 mM IPTG and incubated for an additional 18 h at 20 °C. The cells were harvested by centrifugation and resuspended in a buffer of 20 mM Tris, 10 mM NaCl at pH 8.5 supplemented with 1 mM PMSF. Cells were lysed on ice by ultrasonication, and the lysate was clarified at 4 °C and filtered through a 0.22-μm filter. The supernatant was loaded onto a Q-Sepharose anion-exchange column and washed with a buffer of 20 mM Tris, 10 mM NaCl at pH 8.5. MIF-2 was eluted using a 5% gradient of 20 mM Tris, 1 M NaCl at pH 8.5. MIF-2 was further purified with a 26/60 Superdex 75 column equilibrated with a buffer of 20 mM Tris, 150 mM NaCl at pH 7.4. Protein concentrations were determined by absorbance at 280 nm using ε_280_ = 5500 M^−1^ cm^−1^.

### CD spectroscopy

CD spectra were measured with a Jasco J-815 spectropolarimeter in a 0.2-cm quartz cuvette with 10 μM MIF-2 in a buffer of 20 mM sodium phosphate at pH 7.4. Variable temperature measurements were collected at 218 nm over a temperature range of 25 to 90 °C, sampling 1.5 °C at a rate of 1.5 °C/min.

### Enzymatic assays

4-Hydroxyphenyl pyruvate (4-HPP) at 100 mM in 0.5 M ammonium acetate, pH 6.0, was prepared by overnight agitation at room temperature to generate the keto form. MIF-2 enzymatic activity was determined by monitoring the increase in absorbance at 306 nm caused by enol–borate complex formation between boric acid and 4-HPP in the reaction solution (8). Absorbance was recorded first with a mixture of 1.2 mM 4-HPP and 0.420 M boric acid, then the reaction was initiated by adding MIF-2 at a final concentration of 80 nM, and the absorbance was then again recorded after incubation for 3.5 min. Data were analyzed according to the method of Pantouris *et al.* (23).

### X-ray crystallography

Crystals were grown at 20 °C using the hanging-drop vapor diffusion method and a Formulatrix NT8 robot, with a reservoir volume of 100 μl and drops consisting of 400 nl protein and 200 nl reservoir volume. wt MIF-2 (9.2 mg/ml) was crystallized in 28.6% w/v PEG 3350 and 0.31 M sodium tartrate at pH 5.6 (8). P1G MIF-2 (10.2 mg/ml) was crystallized in 0.5 M ammonium sulfate, 30% v/v (+/−)-2-methyl-2,4-pentanediol, and 0.1 M Hepes at pH 7.5. S62A MIF-2 (13.9 mg/ml) was crystallized in 0.2 N NaCl, 25%w/v PEG 3350, and 0.1 M Tris at pH 8.5 (8). F100A MIF-2 (8.1 mg/ml) was crystallized in 0.2 M ammonium sulfate, 25% w/v PEG 3350, and 0.1 M Tris at pH 8.5. Crystallization was observed with a RI-1000 Formulatrix Imager. Crystals formed in 3 to 20 days.

Diffraction data were collected at 100 K using a Rigaku-007 Micromax Generator with a Pilatus Dectris 200k Pixel Array Detector and AFC 4-axis goniometer. All the datasets were collected and processed with HKL3000 (35). The structures of wt-MIF-2, P1G, S62A, and F100A were solved by molecular replacement using Phaser (36) and chain A of PDB ID 4Q3F or 3KAN as the search models. Refinement was performed in CCP4 using Refmac (37) and PHENIX (38), and manual model building with COOT (39). Data processing and refinement statistics are presented in Table S1. Interfaces were analyzed with Arpeggio (40), and figures were prepared with PyMol (41).

### NMR spectroscopy

NMR experiments were performed on a Bruker Avance NEO 600 MHz spectrometer at 30 °C. NMR data were processed using NMRPipe (42) and analyzed in Sparky (43) along with in-house scripts. Backbone assignments for MIF-2 were obtained by TROSY HNCA, HNCACB, HNCO, HN(CO)CA, and HN(CO)CACB triple-resonance experiments on ^2^H-, ^13^C-, and ^15^N-MIF-2 and deposited into the Biological Magnetic Resonance Bank (entry 50790). NMR assignments of MIF-2 variants were transferred from wt-MIF-2 or confirmed with TROSY HNCA, HNCACB, and HNCO experiments. TROSY-based NMR spin relaxation experiments (44, 45) were performed with the ^1^H and ^15^N carriers set to the water resonance and 120 ppm, respectively. Longitudinal relaxation rates (*R*_*1*_) were measured with *T*_1_ delays of 0, 20, 60, 100, 200, 600, 800, 1200, 1500, 2000, and 2500 ms. Transverse relaxation rates (*R*_*2*_) were collected with 1.0 ms spacing between 180° Carr-Purcell-Meiboom-Gill pulses at total relaxation delays of 0, 16.9, 33.9, 67.8, 136, 169, and 203 ms. The recycle delay in these experiments was 2.5 s. Longitudinal and transverse relaxation rates were extracted by nonlinear least squares fitting of the peak heights to a single exponential decay using in-house software. Uncertainties in these rates were determined from replicate spectra with duplicate relaxation delays of 20, 60, and 600 ms for *T*_1_ and 16.9, 33.9 (x2), 136, and 203 ms for *T*_2_. The heteronuclear cross-relaxation rate (NOE) was obtained by interleaving pulse sequences with and without proton saturation and calculated from the ratio of peak heights from these experiments.

### MD simulations

Seven models of MIF-2 (wt-, F2A, S62A, F100A, F2M, S62H, and F100H) were made using the PDB structure 3KAN (8). The latter three-point mutants (F2M, S62H, and F100H) were chosen to replicate the amino acids present at the equivalent positions in MIF (M2, H62, and Y100). These, as well as the other MIF-2 models, were compared with MIF (PDB ID: 3DJH) (46). For the six MIF-2 mutants, point mutations were made uniformly to all three monomers of MIF-2. All models were solvated with a periodic water box containing sufficient counter ions to neutralize the systems. Simulations were run for 150 ns at 300 K using the NAMD MD software package (47) and the CHARMM36m force field (48), and the equilibrated final 50 ns were analyzed. Analyses were completed using the VMD software package (49). Hydrogen bonds were defined as an acceptor–donor distance of no more than 3.0 Å and an acceptor–donor hydrogen angle no more than 30°. RMSD values and average structures were calculated from the alpha carbons of aligned trajectories. To calculate the number of waters in the central channel of each mutant, the mouth at either end of the channel was defined using the alpha carbons of V42/R42 and Y99/F100 for MIF/MIF-2. The number of waters within these limits was the average for the final 50 ns of the trajectory using 100 frames/ns for a total of 10,000 frames. Occupancy was calculated for central channel waters using the SFALL utility of CCP4 (37, 50, 51, 52). Channel waters from all frames were combined into a single structure file, and a simulated neutron scattering map was created by SFALL at a resolution of 1 Å. To isolate the most consistently occupied positions, the sigma (σ) level was raised to 15 using PyMOL (41).

For the correlation analysis, the last 100 ns of the trajectories were analyzed. The generalized correlation coefficient is defined as $\tilde{r}\left[x_{i},\;x_{j}\right]={\left(1-\exp\left(-\frac{2}{3}\;\tilde{I}\left[x_{i},\;x_{j}\right]\right)\right)}^{\frac{1}{2}}$, where the atomic displacement vectors **x**_k_ are computed from the MD simulations and $\tilde{I}\left[x_{i},\;x_{j}\right]$ is the mutual information between residues *i* and *j*. The mutual information is $\tilde{I}\left[x_{i},\;x_{j}\right]=H\left[x_{i}\right]+H\left[x_{j}\right]-H\left[x_{i},x_{i}\right]$, where $H\left[x_{i}\right]=-\int p\left[x_{i}\right]\ln\left(p\left[x_{\text{i}}\right]\right)dx_{i}$ and $H\left[x_{i},x_{j}\right]=-\iint p\left[x_{i},\;x_{j}\right]\ln\left(p\left[x_{i},\;x_{j}\right]\right)\;dx_{i}dx_{j}$ are the marginal and joint Shannon entropies. The marginal and joint Shannon entropies were computed as ensemble averages over the atomic displacements with marginal and joint probability distributions computed from the thermal fluctuations sampled during the simulations. A more rigorous explanation can be found in other works (53, 54).

### Animals

The wt mice of genetic background strain (C57BL6/J) were purchased from the Jackson Laboratory. Mice were housed at the pathogen-free animal facility at Cooper University Healthcare. All experiments were done in 10- to 12-week-old male mice. Mice were administered a one-time intratracheal instillation of 100 μl of normal saline alone (vehicle) or 10 μg/ml of wt-MIF-2 or one of the MIF-2 allosteric variants: MIF-2–P1G, MIF-2–S62A, or MIF-2–F100A in a normal saline solution. These mice were sacrificed after 6 h of vehicle only or vehicle + experimental agent administration. The BAL fluid was collected for later analysis. The animal study protocol was approved by the Institutional Animal Care and Use Committee of Cooper University Healthcare, Camden, NJ.

### Analysis of the BAL fluid

The BAL fluid was collected 6 h after vehicle only or vehicle + experimental agent administration. BAL collection was performed by cannulating the trachea with a blunt 22-gauge needle and lavaging the lungs with 800 μl of precooled sterile PBS solution. Total cell counts in the BAL fluid were determined using the TC20 automated cell counter (Bio-Rad Laboratories, Inc). The differential cell counts were performed on cells cytocentrifuged onto glass slides (Fisher Scientific) stained with the Hema 3 Staining System (Fisher Diagnostics), and cell differential was tabulated using light microscopy. Total protein concentration in the BAL fluid was measured using the Pierce BCA assay kit (Thermo Scientific), as previously described (55).

## Data availability

X-ray coordinates for wt-MIF-2, P1G, S62A, and F100A have been deposited in the RCSB PDB under accession codes 7MSE, 7MW7, 7MRU, and 7MRV, respectively. Triple resonance NMR assignments for MIF-2 have been deposited in the Biological Magnetic Resonance Bank under ID 50790. All other data related to this work are available upon request to George P. Lisi (george_lisi@brown.edu).

## Supporting information

This article contains supporting information.

## Conflict of interest

The authors declare that they have no conflicts of interest with the contents of this article.
