# Supplementary material for: A structurally preserved allosteric site in the MIF superfamily affects enzymatic activity and CD74 activation in D-dopachrome tautomerase
Source: J Biol Chem. 2021 Aug 9;297(3):101061. doi: 10.1016/j.jbc.2021.101061 (PMC8405996; doi:10.1016/j.jbc.2021.101061)
Supplement: Supplemental Figures S1–S15 and Tables S1–S5 [file mmc1.pdf]

Supporting Information

A Structurally Preserved Allosteric Site in the MIF Superfamily Affects Enzymatic Activity and CD74 Activation in D-dopachrome Tautomerase

Emily Chen,[1] Krystle Reiss,[2] Dilip Shah,[3] Ramu Manjula,[4] Brandon Allen,[2] Eva L. Murphy,[4] James W. Murphy,[4] Victor S. Batista,[2] Vineet Bhandari,[3] Elias J. Lolis,[4]* and George P. Lisi[1]*

[1]Department of Molecular Biology, Cell Biology, & Biochemistry, Brown University, Providence, RI 02903
[2]Department of Chemistry, Yale University, New Haven, CT 06511
[3]Section of Neonatology, Department of Pediatrics, Cooper University Hospital, Camden, NJ 08103
[4]Department of Pharmacology, Yale University School of Medicine, New Haven, CT 06511

*To whom correspondence should be addressed: george_lisi@brown.edu, elias.lolis@yale.edu
Keywords: MIF, allostery, cytokine, CD74, tautomerase

Submitted to J. Biol. Chem.

Table S1. Crystallographic data for wt-MIF-2 and its variants.

	Wild type MIF-2 (PDB:7MSE)	MIF-2 F100A (PDB:7MRV)	MIF-2 S62A (PDB:7MRU)	MIF-2 P1G (PDB:7MW7)
**Data Collection**				
**Wavelength**	1.5418	1.5418	1.5418	1.5418
**Copies in ASU**	3	1	3	1
**Resolution range**	41.7 - 1.27 (1.315 - 1.27)	33.46 - 1.57 (1.627 - 1.57)	42.86 - 1.331 (1.378 - 1.331)	34.58 - 1.1 (1.139 - 1.1)
**Space group**	P 3	R 3:H	P 21 21 21	P 63
**Unit cell**	83.396 83.396 40.429 90 90 120	83.161 83.161 37.781 90 90 120	40.748 75.891 103.862 90 90 90	73.993 73.993 41.067 90.00 90.00 120.00
**Unique reflections**	80431 (5717)	13562 (1323)	70809 (5570)	49994 (3453)
**Redundancy**	11.3 (3.4)	5.2 (3.3)	6.5 (3.7)	10.5 (6.4)
**Completeness (%)**	96.88 (69.08)	99.64 (96.78)	94.74 (75.61)	95.94 (66.57)
**Mean I/sigma(I)**	23.7 (1.95)	48.99 (8.2)	22.06 (2.01)	50.90 (4.08)
**Wilson B-factor**	14.35	13.04	14.21	15.71
**Refinement**				
**Reflections used in refinement**	80424 (5717)	13562 (1323)	70783 (5570)	49981 (3453)
**Reflections used for R-free**	3991 (329)	683 (56)	3481 (266)	2492 (133)
**R-work**	0.1351 (0.1830)	0.1657 (0.1934)	0.1617 (0.2224)	0.1777 (0.2227)
**R-free**	0.1691 (0.2347)	0.2082 (0.2149)	0.1799 (0.2394)	0.1898 (0.2458)
**Number of non-hydrogen atoms**	3208	965	3340	986
**macromolecules**	2738	823	2704	904
**ligands**	30	5	8	6
**solvent**	440	137	628	76
**Protein residues**	351	109	351	118

**RMS (bonds)**	0.012	0.006	0.007	0.007
**RMS (angles)**	1.68	0.87	0.92	0.96
**Ramachandran favored (%)**	97.39	98.13	96.52	96.55
**Ramachandran allowed (%)**	2.61	1.87	3.48	3.45
**Ramachandran outliers (%)**	0.00	0.00	0.00	0.00
**Rotamer outliers (%)**	0.00	0.00	0.00	0.00
**Clash score**	0.72	3.05	3.29	1.09
**Average B-factor**	17.64	14.86	17.12	18.82
**macromolecules**	15.67	13.17	14.83	18.00
**ligands**	21.79	30.43	25.93	22.00
**solvent**	29.61	24.50	26.87	28.26

Table S2. R_1, R_2, and ^{1}H-[^{15}N] NOE values for wt-MIF-2

Residue	*R*1		*R*2		1H-[15N] NOE		Residue	*R*1		*R*2		1H-[15N] NOE	
#	Mean	Std. Dev.	Mean	Std. Dev.	Sat/Unsat	Error	#	Mean	Std. Dev.	Mean	Std. Dev.	Sat/Unsat	Error
2	0.838	0.030	20.833	2.036	0.858	0.045	57	0.880	0.044	20.743	0.362	0.833	0.009
3	0.880	0.061	20.190	1.410	0.856	0.030	59	0.838	0.047	19.186	0.456	0.826	0.018
4	0.820	0.038	18.228	0.704	0.778	0.022	60	0.880	0.055	18.440	0.972	0.807	0.030
5	0.825	0.034	17.867	0.517	0.820	0.019	61	0.820	0.012	18.539	0.344	0.832	0.020
6	0.842	0.011	18.159	0.534	0.844	0.016	62	0.825	0.022	18.868	0.566	0.832	0.029
7	0.861	0.023	19.260	0.275	0.837	0.010	63	0.842	0.017	18.723	1.052	0.803	0.034
8	0.845	0.021	20.321	0.374	0.856	0.012	66	0.867	0.044	18.015	0.266	0.823	0.021
9	0.887	0.037	19.044	0.131	0.861	0.013	68	0.828	0.013	33.727	1.285	0.863	0.035
11	0.824	0.026	19.612	0.143	0.822	0.008	69	0.773	0.022	35.298	2.031	0.856	0.038
12	0.843	0.014	17.541	0.646	0.843	0.026	70	0.861	0.021	19.216	0.447	0.827	0.024
13	0.889	0.110	19.802	0.194	0.812	0.008	71	0.845	0.024	21.959	0.440	0.808	0.014
14	0.826	0.029	17.182	0.133	0.719	0.007	72	0.887	0.039	19.857	0.438	0.834	0.011
16	0.888	0.036	16.639	0.155	0.801	0.009	73	0.888	0.031	19.531	0.108	0.844	0.010
17	1.097	0.118	22.188	2.432	0.713	0.054	74	1.097	0.087	21.925	0.769	0.849	0.023
18	0.921	0.032	20.864	0.250	0.854	0.009	76	0.869	0.016	20.747	1.033	0.847	0.024
19	0.864	0.017	18.443	0.463	0.855	0.018	77	0.849	0.028	18.957	0.385	0.860	0.022
20	0.847	0.022	19.790	0.374	0.786	0.016	79	0.921	0.022	19.391	0.530	0.840	0.017
21	0.917	0.028	18.172	0.717	0.838	0.024	80	0.864	0.029	19.912	1.142	0.812	0.023
22	0.882	0.023	20.362	0.316	0.880	0.018	81	0.847	0.021	19.440	0.775	0.845	0.019
23	0.894	0.014	19.916	0.341	0.829	0.018	82	0.815	0.011	18.086	0.226	0.835	0.013
24	0.869	0.034	20.721	0.167	0.839	0.011	83	0.862	0.020	19.109	0.110	0.840	0.011
25	0.889	0.031	20.760	0.309	0.846	0.007	84	0.917	0.023	19.124	0.240	0.843	0.008
26	0.862	0.029	19.924	0.263	0.860	0.010	85	0.882	0.029	24.091	0.529	0.822	0.020
27	0.867	0.025	21.146	0.271	0.837	0.020	86	0.894	0.021	18.811	0.222	0.848	0.013
28	0.842	0.013	18.936	0.441	0.816	0.013	87	0.869	0.023	18.961	0.281	0.822	0.015
29	0.877	0.020	17.010	0.324	0.775	0.026	88	0.889	0.020	17.268	0.423	0.832	0.012
30	0.858	0.035	17.581	0.760	0.854	0.034	89	0.862	0.032	20.610	0.291	0.849	0.010
31	0.846	0.026	18.365	0.310	0.797	0.021	90	0.867	0.015	17.265	0.358	0.867	0.018
32	0.828	0.023	21.552	0.382	0.799	0.023	91	0.842	0.019	17.771	0.309	0.835	0.010
34	0.848	0.016	19.956	0.266	0.751	0.012	92	0.877	0.034	18.829	0.251	0.810	0.013
35	0.871	0.042	28.361	1.351	0.765	0.028	93	0.858	0.040	21.368	0.267	0.822	0.015
36	0.865	0.029	20.032	1.192	0.813	0.025	94	0.846	0.032	18.968	0.165	0.842	0.009
37	0.850	0.019	18.342	1.083	0.656	0.028	95	0.828	0.032	20.408	0.118	0.851	0.009
38	0.898	0.037	14.914	2.380			96	0.848	0.017	19.829	0.297	0.864	0.013
39	0.840	0.030	19.212	0.151	0.851	0.016	97	0.871	0.041	19.658	0.359	0.858	0.016
40	0.859	0.015	18.501	0.479	0.849	0.016	98	0.865	0.033	18.904	0.230	0.855	0.019
41	0.850	0.025	20.080	0.480	0.860	0.014	99	0.850	0.024	20.313	0.953	0.836	0.024
42	0.826	0.022	19.172	0.216	0.862	0.013	100	0.898	0.054	18.975	0.983	0.853	0.020
44	0.824	0.033	19.822	0.253	0.849	0.013	103	0.885	0.049	19.406	0.674	0.814	0.023
45	0.869	0.019	21.556	0.711	0.851	0.011	104	0.960	0.036	18.225	0.212	0.835	0.015
46	0.815	0.024	19.091	0.234	0.849	0.014	106	0.887	0.020	20.929	0.393	0.826	0.029
47	0.815	0.013	18.907	0.345	0.858	0.014	107	0.953	0.027	28.281	1.848	0.886	0.029
48	0.822	0.030	18.646	1.314	0.842	0.023	110	0.866	0.015	20.263	0.328	0.839	0.017
49	0.885	0.019	17.775	0.404	0.828	0.019	113	0.875	0.021	20.408	0.658	0.866	0.021
50	0.953	0.011	18.608	0.582	0.825	0.024	114	0.874	0.030	19.066	0.333	0.860	0.012
51	0.889	0.097	20.149	0.318	0.807	0.020	115	0.785	0.031	19.992	1.039	0.928	0.042
52	0.901	0.030	17.358	0.253	0.810	0.012	116	0.933	0.026	20.450	1.330	0.871	0.038
54	0.822	0.018	20.492	0.142	0.847	0.011	117	0.935	0.042	20.747	0.215	0.849	0.012
56	0.857	0.013	20.500	0.551	0.847	0.012							

Table S3. R_1, R_2, and ^{1}H-[^{15}N] NOE values for P1G MIF-2

Residue	*R1*		*R2*		1H-[15N] NOE		Residue	*R1*		*R2*		1H-[15N] NOE	
#	Mean	Std. Dev.	Mean	Std. Dev.	Sat/Unsat	Error	#	Mean	Std. Dev.	Mean	Std. Dev.	Sat/Unsat	Error
2	0.858	0.038	19.716	0.902	0.820	0.014	57	0.854	0.022	19.554	0.382	0.853	0.008
3	0.935	0.023	19.497	0.167	0.838	0.008	59	0.931	0.026	20.400	0.301	0.863	0.006
4	0.832	0.023	18.914	0.141	0.827	0.007	60	0.839	0.017	18.308	0.506	0.845	0.009
5	0.822	0.011	19.301	0.323	0.842	0.008	61	0.865	0.033	18.657	0.207	0.842	0.007
6	0.845	0.020	19.033	0.178	0.851	0.007	63	0.895	0.037	21.482	0.459	0.729	0.014
7	0.842	0.033	18.779	0.164	0.858	0.005	66	0.847	0.032	19.853	0.548	0.847	0.014
8	0.846	0.023	19.952	0.274	0.848	0.006	69	0.890	0.020	22.467	0.586	0.847	0.010
9	0.887	0.037	19.242	0.325	0.851	0.007	70	0.913	0.024	19.286	0.113	0.834	0.007
11	0.838	0.027	19.778	0.123	0.827	0.005	71	0.873	0.030	18.947	0.093	0.848	0.005
12	0.887	0.024	17.848	0.516	0.852	0.013	72	0.889	0.028	19.260	0.303	0.835	0.008
13	0.824	0.025	19.520	0.299	0.802	0.005	73	0.846	0.012	20.288	0.232	0.867	0.010
14	0.787	0.017	17.525	0.189	0.737	0.004	76	0.787	0.041	14.524	2.086	0.863	0.057
16	0.869	0.032	16.835	0.198	0.825	0.005	77	0.890	0.047	18.929	0.248	0.832	0.007
17	0.841	0.031	17.053	0.512	0.809	0.023	79	0.863	0.018	18.584	0.473	0.850	0.006
18	0.919	0.016	20.272	0.167	0.873	0.005	80	0.885	0.014	20.313	0.271	0.865	0.007
19	0.836	0.023	18.751	0.506	0.833	0.007	81	0.881	0.017	20.040	0.122	0.858	0.007
20	0.847	0.022	18.829	0.147	0.789	0.006	82	0.847	0.030	20.109	0.154	0.835	0.005
21	0.872	0.025	20.942	0.332	0.817	0.005	83	0.850	0.029	20.040	0.118	0.842	0.004
22	0.882	0.017	20.012	0.573	0.823	0.008	84	0.853	0.028	18.598	0.184	0.847	0.004
23	0.867	0.012	20.890	0.425	0.836	0.005	85	0.865	0.030	21.182	0.446	0.858	0.007
24	0.878	0.038	19.227	0.233	0.836	0.006	86	0.833	0.014	19.673	0.134	0.838	0.005
25	0.883	0.033	19.932	0.205	0.849	0.005	87	0.831	0.009	18.843	0.158	0.833	0.006
26	0.862	0.018	19.806	0.068	0.840	0.006	88	0.826	0.013	17.982	0.705	0.835	0.006
27	0.880	0.028	19.440	0.231	0.831	0.008	89	0.845	0.021	20.492	0.207	0.852	0.005
28	0.826	0.017	18.051	0.485	0.829	0.008	90	0.767	0.008	17.053	0.197	0.830	0.005
29	0.852	0.040	21.286	0.557	0.816	0.015	91	0.816	0.028	18.365	0.221	0.837	0.006
30	0.864	0.023	18.060	0.457	0.862	0.014	92	0.913	0.009	18.871	0.295	0.815	0.005
31	0.864	0.025	18.175	0.380	0.845	0.011	93	0.914	0.012	20.113	0.121	0.823	0.006
32	0.847	0.023	18.699	0.692	0.772	0.010	94	0.861	0.014	19.117	0.211	0.856	0.006
34	0.855	0.018	19.168	0.266	0.776	0.005	95	0.863	0.026	19.837	0.043	0.856	0.005
35	0.854	0.041	18.437	0.935	0.694	0.008	96	0.853	0.033	19.577	0.126	0.851	0.006
37	0.858	0.023	18.423	0.145	0.772	0.008	97	0.854	0.020	17.388	1.218	0.805	0.014
38	0.745	0.084	4.425	3.230	0.820	0.011	98	0.870	0.013	19.073	0.358	0.844	0.011
39	0.845	0.021	19.124	0.567	0.862	0.007	99	0.823	0.026	20.859	0.305	0.859	0.010
40	0.847	0.026	18.918	0.157	0.843	0.006	100	0.824	0.021	18.961	0.236	0.856	0.010
41	0.825	0.034	19.139	0.103	0.851	0.006	103	0.808	0.009	19.585	0.598	0.834	0.014
42	0.833	0.021	19.205	0.210	0.839	0.006	104	0.874	0.036	18.440	0.144	0.820	0.009
44	0.833	0.012	19.837	0.230	0.851	0.006	106	0.916	0.041	20.760	0.344	0.868	0.008
45	0.854	0.024	21.204	0.347	0.847	0.004	107	0.837	0.020	25.044	1.825	0.892	0.017
46	0.831	0.016	20.572	0.461	0.847	0.006	110	0.794	0.035	16.878	1.692	0.808	0.030
47	0.815	0.012	18.681	0.175	0.840	0.007	111	0.898	0.020	20.799	0.869	0.815	0.012
48	0.838	0.007	19.554	0.215	0.826	0.006	113	0.886	0.024	19.822	0.273	0.856	0.008
49	0.855	0.022	18.532	0.213	0.775	0.008	114	0.883	0.045	18.570	0.441	0.873	0.009
52	0.912	0.021	16.975	0.067	0.779	0.004	115	0.894	0.053	18.716	0.427	0.850	0.019
54	0.907	0.014	20.113	0.103	0.833	0.006	116	0.838	0.017	19.736	0.248	0.845	0.010
56	0.873	0.024	20.247	0.311	0.862	0.006	117	0.907	0.026	19.944	0.082	0.860	0.005

Table S4. R_1, R_2, and ^{1}H-[^{15}N] NOE values for S62A MIF-2

Residue	*R*1		*R*2		1H-[15N] NOE		Residue	*R*1		*R*2		1H-[15N] NOE	
#	Mean	Std. Dev.	Mean	Std. Dev.	Sat/Unsat	Error	#	Mean	Std. Dev.	Mean	Std. Dev.	Sat/Unsat	Error
2	0.816	0.036	20.068	1.760	0.865	0.048	56	0.878	0.019	21.245	0.221	0.837	0.015
3	0.868	0.032	19.474	0.451	0.835	0.027	57	0.864	0.014	21.106	0.092	0.831	0.009
4	0.845	0.027	19.429	0.502	0.832	0.017	59	0.813	0.049	19.497	1.775	0.969	0.076
5	0.886	0.039	17.921	1.195	0.903	0.024	60	0.890	0.018	19.106	0.342	0.851	0.023
6	0.871	0.025	17.883	0.169	0.830	0.017	61	0.835	0.023	20.088	0.670	0.859	0.015
7	0.852	0.012	19.708	0.185	0.846	0.011	66	0.816	0.034	21.617	0.701	0.746	0.049
8	0.854	0.018	20.589	0.342	0.870	0.011	68	0.994	0.047	42.355	5.472	0.762	0.046
9	0.869	0.011	19.972	0.339	0.844	0.013	69	0.848	0.125	35.063	3.221	0.762	0.044
11	0.857	0.023	19.865	0.157	0.833	0.008	70	0.930	0.031	18.598	0.699	0.791	0.025
12	0.917	0.038	19.120	0.632	0.847	0.021	71	0.915	0.027	22.462	0.273	0.820	0.017
13	0.826	0.021	19.681	0.205	0.809	0.006	72	0.920	0.029	20.080	0.218	0.825	0.012
14	0.804	0.015	17.446	0.131	0.724	0.007	73	0.941	0.014	19.763	0.293	0.875	0.013
16	0.883	0.014	17.203	0.214	0.650	0.009	74	0.977	0.036	23.929	1.054	0.816	0.025
17	1.078	0.056	18.657	1.107	0.851	0.057	76	0.923	0.040	20.704	0.493	0.854	0.024
18	0.934	0.030	21.245	0.202	0.841	0.010	77	0.888	0.023	20.121	0.822	0.844	0.023
19	0.880	0.013	20.036	0.324	0.843	0.017	79	0.884	0.019	20.092	0.597	0.821	0.018
20	0.858	0.025	19.673	0.403	0.788	0.017	80	0.895	0.063	19.739	0.655	0.815	0.029
21	0.919	0.026	19.739	0.803	0.830	0.019	81	0.873	0.020	22.022	0.757	0.872	0.022
22	0.894	0.036	21.017	0.292	0.845	0.015	82	0.927	0.022	11.016	0.822	0.680	0.013
23	0.878	0.018	19.980	0.583	0.826	0.017	83	0.862	0.009	20.425	0.171	0.811	0.014
24	0.875	0.014	20.563	0.250	0.846	0.014	84	0.839	0.022	18.882	0.328	0.847	0.009
25	0.890	0.026	20.964	0.122	0.821	0.008	85	0.925	0.046	22.523	0.619	0.828	0.023
26	0.866	0.033	20.141	0.099	0.844	0.011	86	0.815	0.012	20.080	0.215	0.820	0.012
27	0.884	0.024	21.195	0.295	0.852	0.016	87	0.850	0.005	19.712	0.212	0.835	0.015
28	0.818	0.014	19.654	1.537	0.843	0.012	88	0.812	0.018	18.162	0.459	0.830	0.013
29	0.883	0.027	18.423	0.441	0.758	0.025	89	0.837	0.023	20.627	0.213	0.834	0.011
30	0.838	0.012	19.417	0.615	0.831	0.029	90	0.788	0.027	17.516	0.328	0.824	0.017
31	0.878	0.039	19.190	0.818	0.822	0.021	91	0.820	0.028	18.389	0.289	0.825	0.013
32	0.826	0.022	20.020	0.271	0.875	0.024	92	0.903	0.061	19.135	0.421	0.800	0.013
34	0.869	0.023	21.863	0.426	0.779	0.011	93	0.935	0.026	21.848	0.649	0.840	0.018
35	0.847	0.031	25.628	1.077	0.700	0.031	94	0.858	0.013	19.264	0.123	0.850	0.009
36	0.870	0.022	20.708	0.428	0.774	0.021	95	0.869	0.031	21.008	0.125	0.852	0.010
37	0.851	0.018	18.734	0.330	0.715	0.033	96	0.845	0.016	20.088	0.228	0.854	0.014
38	0.841	0.120	8.157	0.892			97	0.840	0.016	20.467	0.245	0.809	0.014
39	0.845	0.022	19.095	0.536	0.831	0.019	98	0.855	0.028	19.264	0.460	0.826	0.025
40	0.850	0.029	18.295	0.361	0.842	0.018	99	0.800	0.033	22.065	1.300	0.866	0.032
41	0.803	0.026	18.136	0.585	0.901	0.017	100	0.828	0.011	19.724	0.494	0.837	0.028
42	0.830	0.016	20.674	0.774	0.855	0.024	103	0.814	0.088	20.576	1.211	0.815	0.030
44	0.801	0.013	19.600	0.473	0.831	0.014	104	0.925	0.041	18.553	0.506	0.840	0.019
45	0.870	0.040	20.589	0.445	0.844	0.010	106	0.896	0.026	20.500	0.958	0.826	0.037
46	0.799	0.017	19.984	0.284	0.828	0.014	107	0.814	0.030	28.827	0.856	0.885	0.034
47	0.835	0.029	19.272	0.195	0.850	0.014	110	0.859	0.016	20.563	0.516	0.808	0.023
48	0.817	0.025	21.173	0.328	0.836	0.024	111	0.912	0.075	33.080	5.931	0.732	0.064
49	0.887	0.028	18.822	0.386	0.845	0.021	112	0.811	0.031	18.943	1.112	0.877	0.052
50	0.943	0.026	19.357	0.420	0.822	0.023	113	0.885	0.023	21.459	0.907	0.813	0.019
51	0.928	0.042	19.059	0.498	0.812	0.020	114	0.907	0.029	20.129	0.363	0.855	0.017
52	0.901	0.020	18.508	0.259	0.822	0.010	116	1.000	0.063	18.051	1.160	0.885	0.046
54	0.919	0.034	20.747	0.076	0.857	0.013	117	0.962	0.042	21.115	0.236	0.824	0.012

Table S5. R_1, R_2, and ^{1}H-[^{15}N] NOE values for F100A MIF-2

Residue	*R*1		*R*2		Residue	1H-[15N] NOE		Residue	*R*1		*R*2		Residue	1H-[15N] NOE	
#	Mean	SD	Mean	SD	#	Sat/Unsat	Error	#	Mean	SD	Mean	SD	#	Sat/Unsat	Error
2	0.897	0.033	18.342	1.524	2	0.901	0.072	47	0.784	0.038	19.410	0.266	50	0.770	0.043
3	0.791	0.026	21.901	1.588	3	0.850	0.032	48	0.895	0.079	20.169	1.440	52	0.812	0.016
4	0.854	0.057	20.333	0.736	4	0.815	0.029	49	0.824	0.030	23.929	1.237	54	0.858	0.023
5	0.801	0.014	19.040	0.805	5	0.871	0.035	50	0.882	0.049	20.964	1.635	56	0.841	0.023
6	0.835	0.042	19.414	0.558	6	0.828	0.036	52	0.865	0.025	18.195	0.156	57	0.816	0.012
7	0.814	0.032	19.829	0.203	7	0.844	0.020	54	0.921	0.018	20.222	0.638	59	0.893	0.064
8	0.826	0.020	21.349	0.675	8	0.869	0.022	56	0.844	0.038	22.558	0.763	60	0.875	0.053
9	0.869	0.018	19.592	0.384	9	0.841	0.021	57	0.817	0.026	24.260	0.550	70	0.849	0.033
11	0.826	0.027	20.868	0.217	11	0.840	0.010	59	1.031	0.135	23.403	1.205	71	0.883	0.057
12	0.928	0.096	19.361	0.690	12	0.826	0.024	60	0.833	0.039	23.529	1.921	72	0.783	0.031
13	0.792	0.024	20.182	0.140	13	0.798	0.008	70	0.929	0.136	22.904	0.761	73	0.795	0.071
14	0.760	0.031	18.073	0.219	14	0.713	0.010	72	0.907	0.062	21.200	0.791	76	0.924	0.050
16	0.858	0.039	17.947	0.102	16	0.795	0.014	73	0.853	0.071	24.795	2.834	77	0.851	0.077
18	0.880	0.023	21.404	0.268	17	0.884	0.075	76	0.825	0.038	18.727	1.364	79	0.798	0.052
19	0.799	0.018	20.044	0.571	18	0.862	0.018	79	0.984	0.127	22.578	3.619	81	0.909	0.062
20	0.873	0.064	21.608	0.859	19	0.902	0.051	82	0.960	0.023	14.639	0.949	82	0.800	0.035
21	0.878	0.018	19.142	0.824	20	0.790	0.031	83	0.838	0.032	20.396	1.073	83	0.826	0.028
22	0.896	0.037	21.598	0.849	21	0.831	0.019	84	0.847	0.041	19.505	0.609	84	0.853	0.018
23	0.845	0.030	20.665	0.478	22	0.868	0.025	86	0.859	0.028	20.509	0.564	86	0.851	0.031
24	0.862	0.023	21.000	0.279	23	0.832	0.021	87	0.844	0.027	20.956	0.501	87	0.821	0.023
25	0.847	0.034	22.099	0.874	24	0.814	0.023	88	0.796	0.027	18.403	0.188	88	0.839	0.023
26	0.867	0.042	19.996	0.193	25	0.798	0.019	89	0.833	0.029	21.725	0.415	89	0.850	0.025
27	0.894	0.039	20.317	0.454	26	0.858	0.021	90	0.708	0.055	18.737	0.983	90	0.705	0.038
28	0.810	0.020	19.493	0.179	27	0.860	0.022	91	0.861	0.037	18.950	0.711	91	0.807	0.027
29	1.231	0.046	7.364	0.759	28	0.776	0.018	92	0.928	0.037	20.272	1.352	92	0.739	0.031
30	0.829	0.027	17.322	0.723	30	0.875	0.033	94	0.831	0.029	19.908	0.290	93	0.869	0.065
31	0.816	0.025	19.619	0.570	31	0.798	0.045	95	0.870	0.050	21.887	0.604	94	0.839	0.015
32	0.820	0.029	19.759	0.535	32	0.758	0.032	96	0.896	0.038	24.325	2.716	95	0.843	0.020
34	0.863	0.036	22.983	0.141	34	0.746	0.015	97	0.829	0.024	20.700	0.883	96	0.825	0.039
35	0.802	0.031	28.169	1.389	35	0.740	0.047	98	0.962	0.093	21.978	1.241	97	0.801	0.020
36	0.845	0.044	20.492	0.613	36	0.769	0.028	103	0.959	0.175	17.908	1.097	98	0.768	0.044
37	0.804	0.050	19.531	1.114	37	0.675	0.037	104	0.964	0.026	21.935	0.601	103	0.806	0.045
38	0.635	0.056	6.285	2.908	39	0.876	0.049	107	0.957	0.108	43.328	3.529	104	0.804	0.040
39	0.925	0.082	20.342	1.051	40	0.841	0.030	110	0.798	0.052	24.845	3.808	107	0.825	0.055
40	0.837	0.044	21.858	0.822	41	0.873	0.022	112	0.901	0.055	22.624	2.303	110	0.797	0.065
41	0.802	0.032	21.057	0.457	42	0.868	0.023	113	0.980	0.054	36.657	3.924	111	0.911	0.093
42	0.791	0.006	20.751	0.391	44	0.801	0.027	114	1.001	0.088	28.977	0.698	112	0.863	0.073
44	0.833	0.023	20.816	0.416	45	0.818	0.019	115	0.912	0.142	28.209	4.178	113	0.798	0.058
45	0.856	0.047	20.392	0.373	46	0.804	0.034	117	1.021	0.135	19.685	0.628	114	0.891	0.042
46	0.751	0.069	22.331	2.688	47	0.837	0.027						115	0.928	0.086
					48	0.832	0.045						117	0.755	0.034
					49	0.923	0.041								

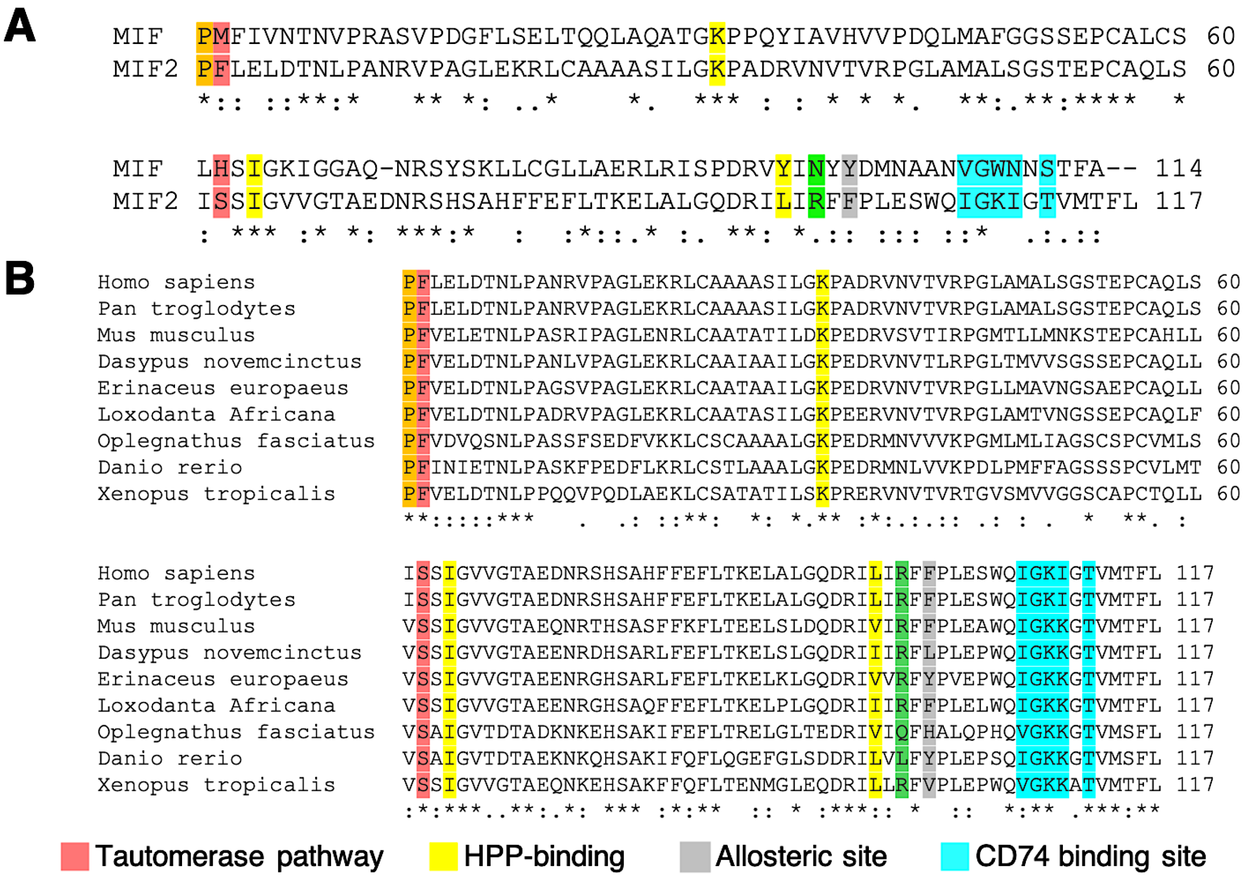

Figure S1. Multiple sequence alignment of MIF proteins. **(A)** Sequence alignment of human MIF (top) and MIF-2 (bottom). **(B)** Sequence alignment to assess the conservation of amino acids in MIF-2 across various species. In both **(A)** and **(B)**, residues corresponding to the enzymatic active site (red), ligand binding site (yellow), allosteric solvent channel pathway (gray), and C-terminal CD74 activation site (cyan) are colored according to the included legend. Residues that are fully conserved are indicated by stars, residues that are highly similar are indicated with two dots, and residues that are similar are indicated with single dots. Of particular interest; residues corresponding to the allosteric sites and CD74 activation site are not conserved between MIF and MIF-2, but are conserved among MIF-2 species.

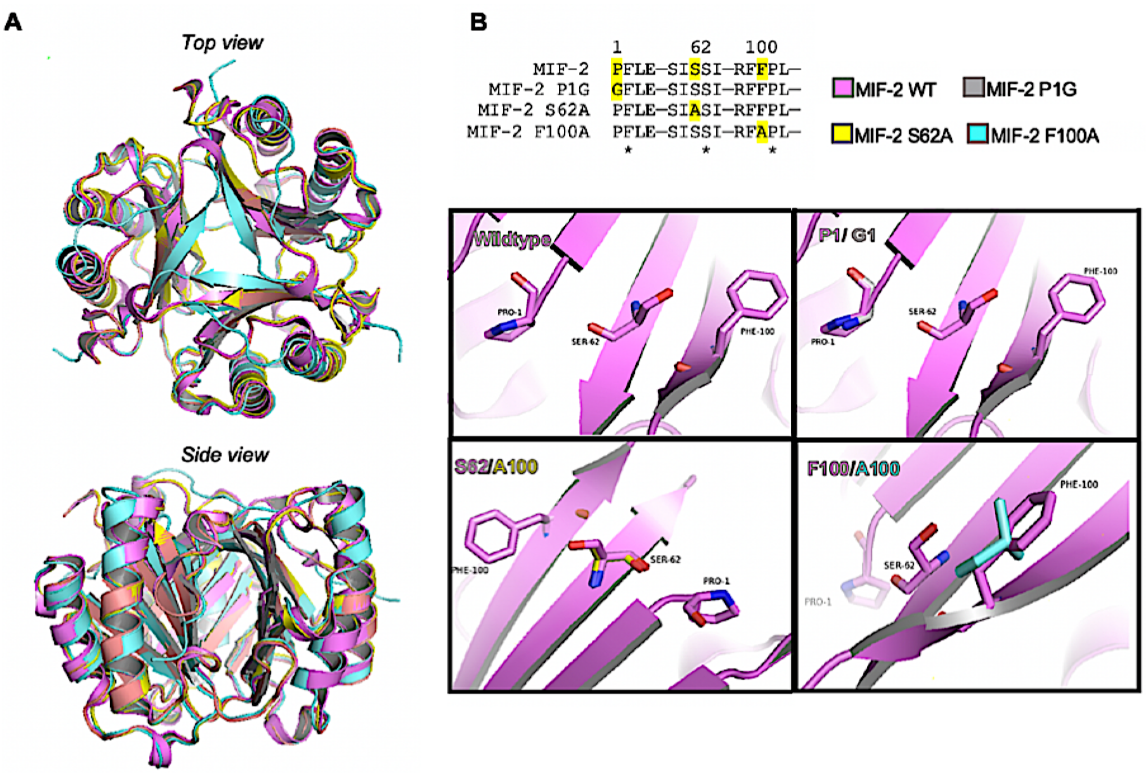

Figure S2. X-ray crystallography of wt-MIF-2 and allosteric variants. **(A)** Crystal structure overlay of MIF-2 (pink), MIF-2 P1G (gray), MIF-2 S62A (yellow), and MIF-2 F100A (teal). **(B)** Sequence alignment of mutated amino acids and the side chain orientation of the mutated residue overlaid with that of the wt-MIF-2 side chain.

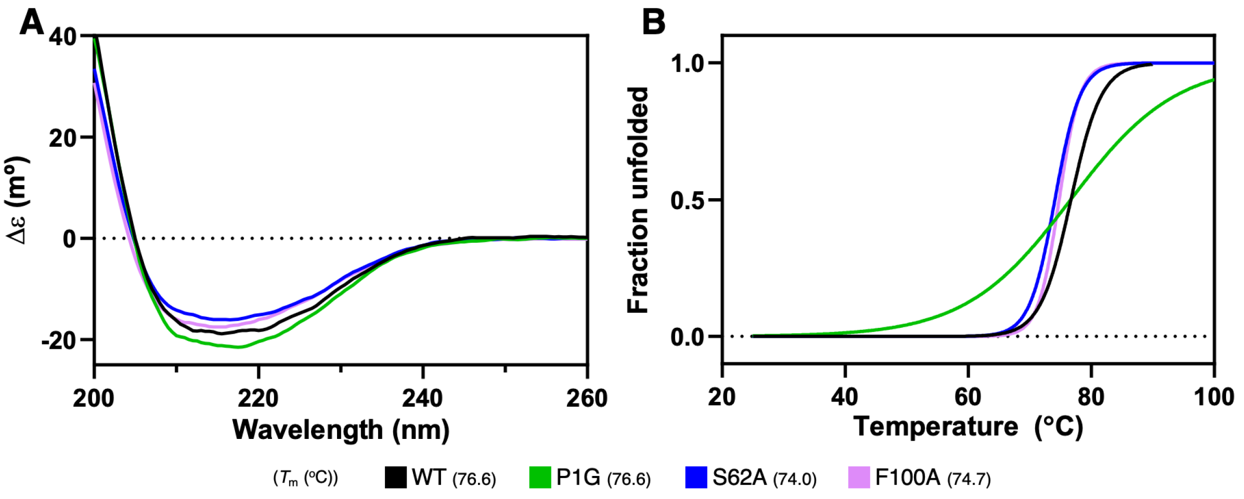

Figure S3. Secondary structure and stability of MIF-2 variants. **(A)** Far-UV circular dichroism spectroscopy showing the α-β secondary structure is retained upon mutation of allosteric residues **(B)** Assessment of thermal stability of MIF variants (λ = 218 nm) showing that thermal stabilities, indicated at bottom according to the legend, are generally similar upon mutation of allosteric residues. Pro1 mutants are well known to produce broad unfolding profiles that modulate the sharp two-state transition of wt-MIF and wt-MIF-2. Solid lines represent best fits of the data.

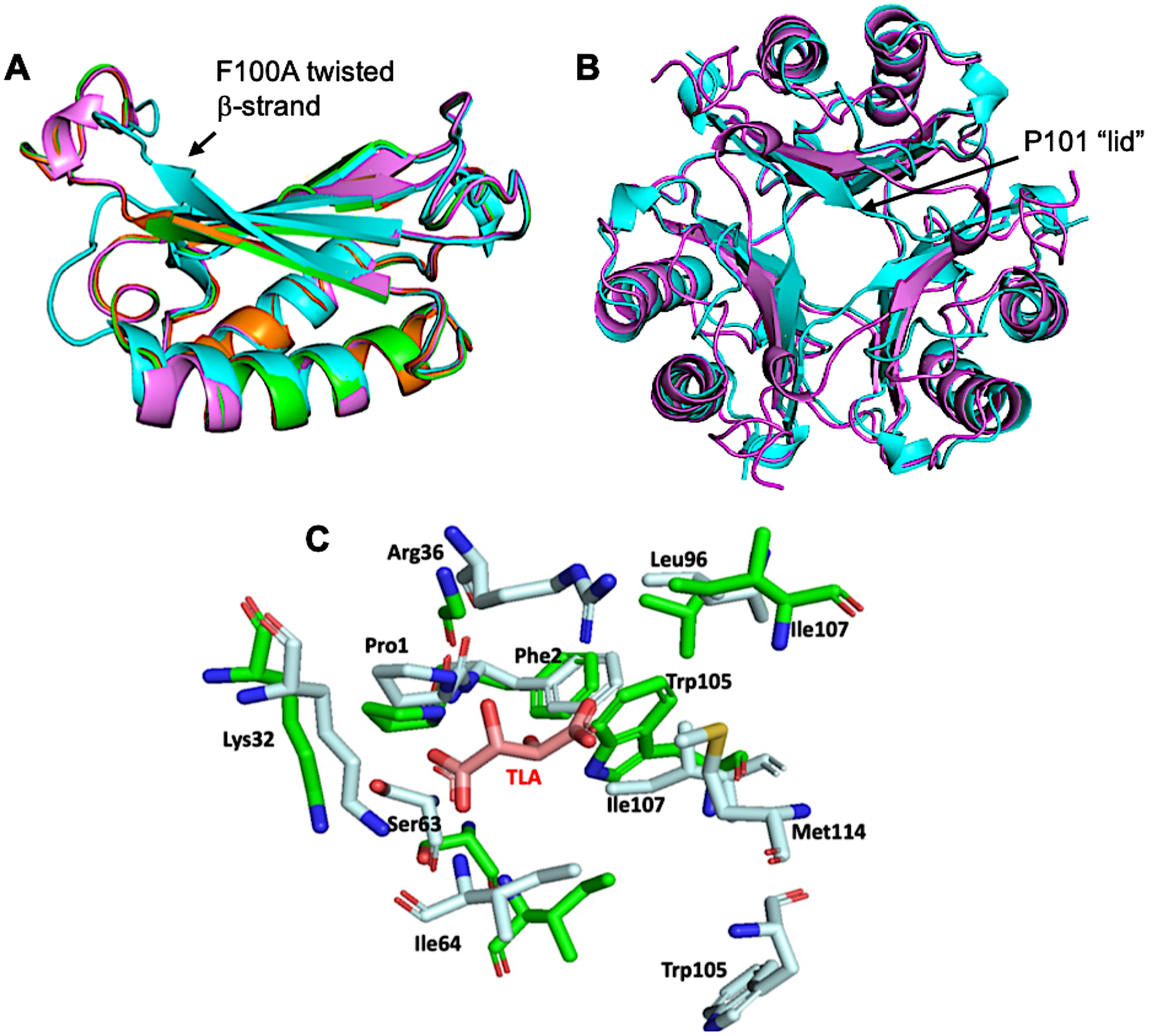

Figure S4. Overlaid wt-MIF-2 and variant monomers, wt-MIF-2 and F100A trimers and active sites based on X-ray crystallography. **(A)** The overlaid monomers show that wt-MIF-2 (magenta) and variants P1G (orange) and S62A (green) have a tight overlap, but F100A (cyan) has a twisted β-strand, and a loop (mid left) and C-terminal region (top left) that are displaced. **(B)** The wt-MIF-2 and F100A trimers show how these changes in the displaced F100A β-strands from each subunit come closer together create a lid on the solvent cavity via three Pro101 residues (also see **Fig. 3C, D**). **(C)** Residues of wt-MIF-2 with atoms within 4 Å of the tartrate molecule (TLA, from the crystallization conditions) in the active site (light blue carbon atoms) superimposed on the F100A variant. The F100A variant has sulfate instead of tartrate, which is not in the crystallization conditions for F100A, in every active site. For comparison, the sulfate was removed and the wt- and F100A (green carbon atoms) were aligned for examination of the active sites. There is no electron density for the side chain of Arg36 and for the entire region from residues 110-117 including the active site Met114. The space from additional movement of Ile107 in the F100A variant from its wt position is filled by Trp105, caused by the displacement of this truncated C-terminal region to reconstitute an enzymatic site with limited activity.

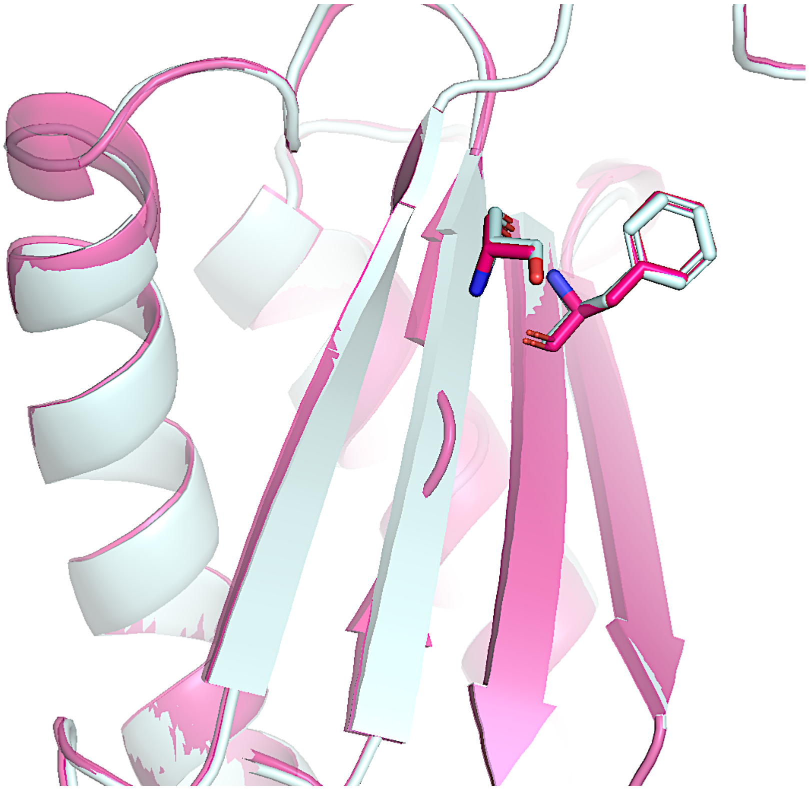

Figure S5. Effect of Ser62 mutation on the Phe2 side chain. Despite Ser62 being in close proximity to Phe2, an S62A mutation (light blue) does not alter the position of the Phe2 side chain when compared to wt-MIF-2 (pink) in X-ray crystal structures.

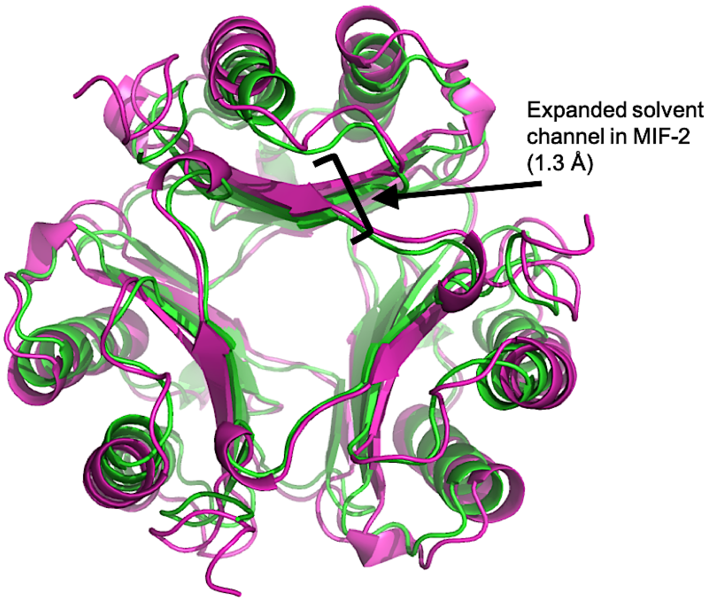

Figure S6. Expanded solvent channel of MIF-2 captured during MD simulations. The MIF trimer is shown in green, while MIF-2 is shown in magenta. **NOTE:** X-ray crystallographic data comparing wt-MIF-2 and MIF-2 variants show changes in the solvent channel opening, which are distinct from those shown here, since this Figure compares the MIF and MIF-2 proteins.

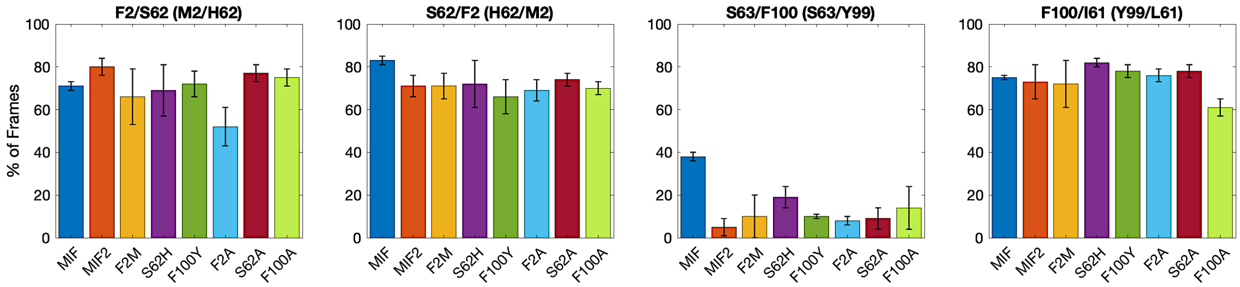

Figure S7. Inter-beta strand hydrogen bonds connecting the Pro-1 active site to Y99/F100 for wt-MIF and wt-MIF2, as well as MIF-2 variants. Each interaction is labeled as the MIF-2 donor/acceptor pair, with the equivalent MIF donor/acceptor pair in parentheses. A hydrogen bond is defined here as a donor-acceptor distance of ≤3.0 Å and an acceptor-donor-hydrogen angle of ≤30°. Values shown are the average of the three monomers (± standard deviation).

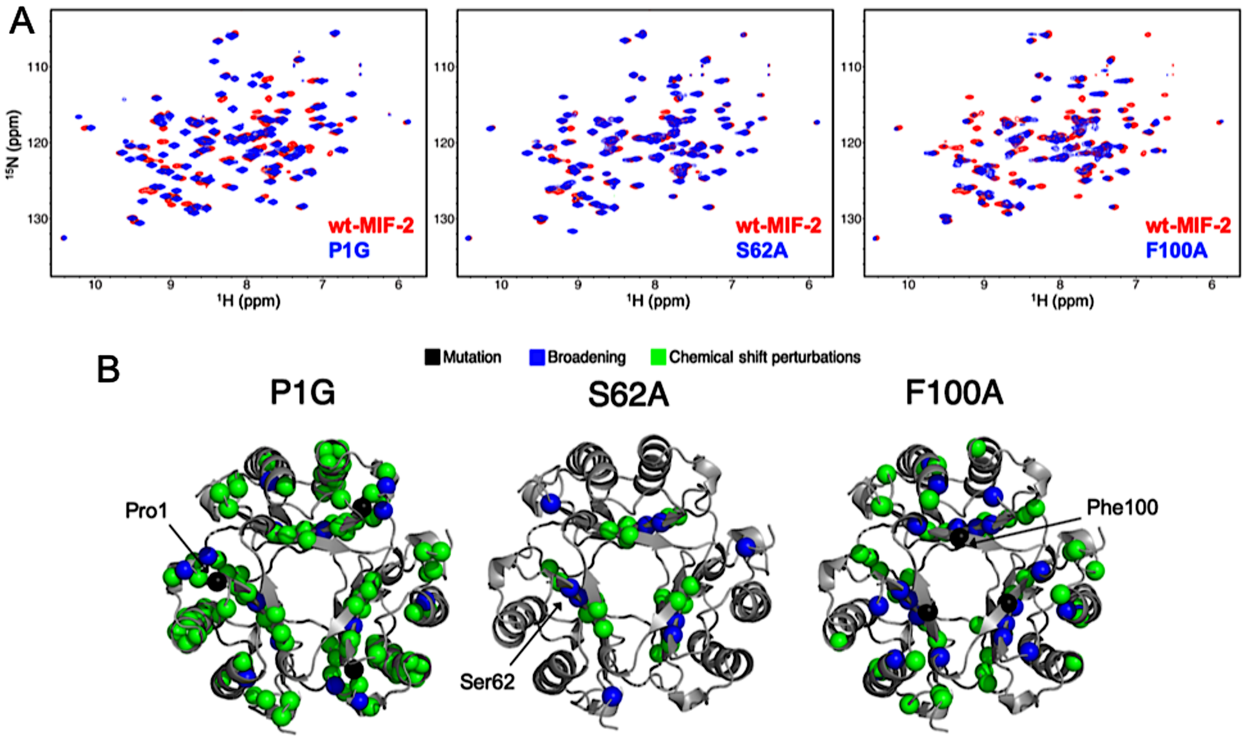

Figure S8. (A) ^{1}H-^{15}N TROSY-HSQC NMR spectral overlays of wt-MIF-2 (red) and allosteric variants (blue). **(B)** Summary of NMR spectral perturbations caused by mutations in MIF-2, where chemical shift perturbations (green spheres, defined as 1.5σ above the 10% trimmed mean of all shifts, as in **Figure 5A**) and sites of line broadening (blue spheres; defined as a loss of ≥50% of wt- resonance intensity) are mapped onto the MIF-2 trimer. Sites of mutation are indicated by black spheres and arrows.

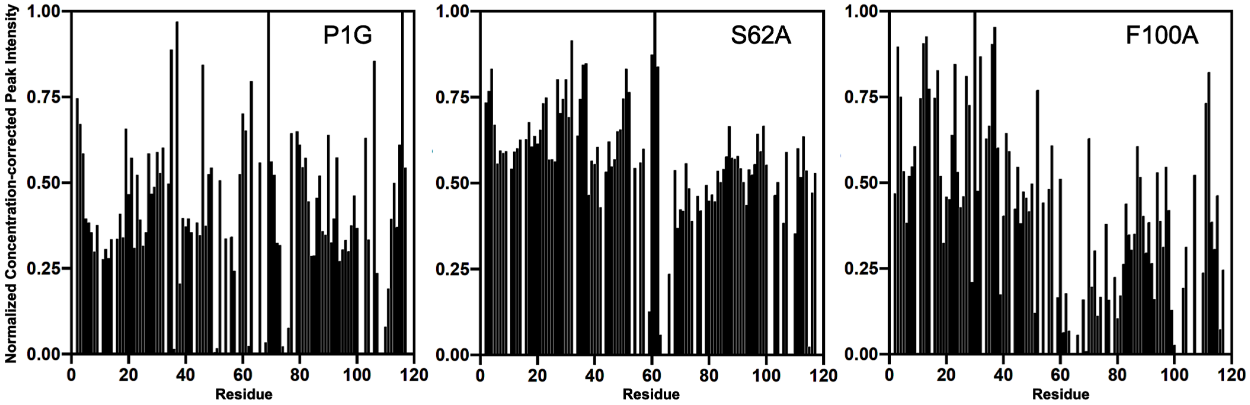

Figure S9. Normalized, concentration-corrected NMR resonance intensity changes caused by MIF-2 variants. wt-MIF-2 and MIF-2 variant peak intensities were recorded with Sparky's Peak Height Analysis tool. Peak intensities of MIF-2 variants were corrected for concentration by $Intensity_{(mut)}$ / $Intensity_{(wt)}$ based on known protein concentrations. These values were then divided by the peak intensities of wt-MIF-2 and normalized against the maximum intensity value for each variant.

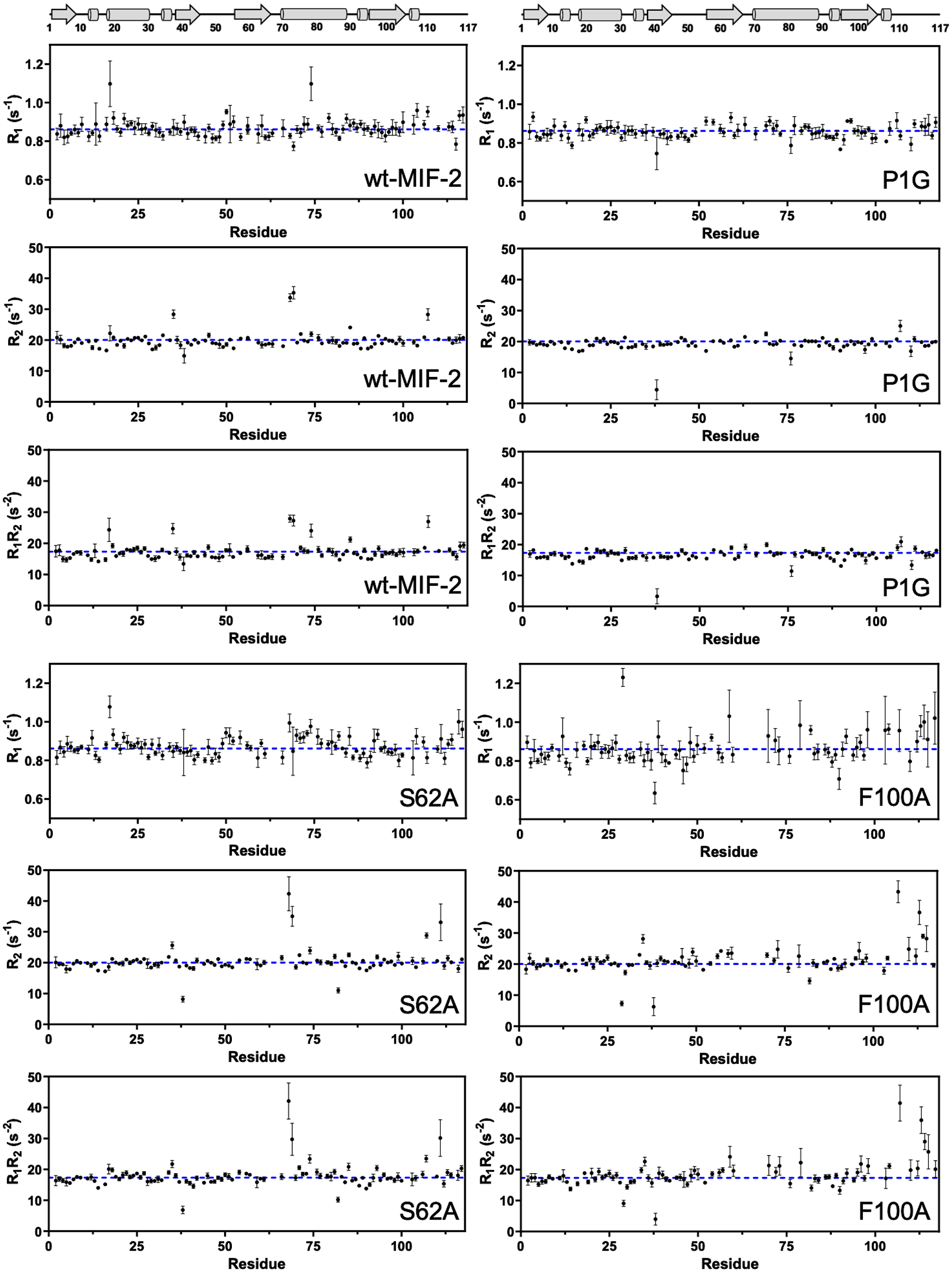

Figure S10. Summary of NMR R_1 and R_2 relaxation parameters. The per-residue R_1, R_2, and R_1R_2 values are plotted for wt-MIF-2 and variants P1G, S62A, and F100A. Overall, the plots of the mutants are relatively similar to wt-MIF-2, though local differences can be observed. Most notably, there are significant dynamic changes at the C-terminal CD74 activation region caused by an F100A mutation, suggesting a coupling between these two sites. Blue dashed lines represent the 10% trimmed mean of all relaxation rates for a given parameter. Cartoons at top show the secondary structure elements of MIF-2 (not to scale).

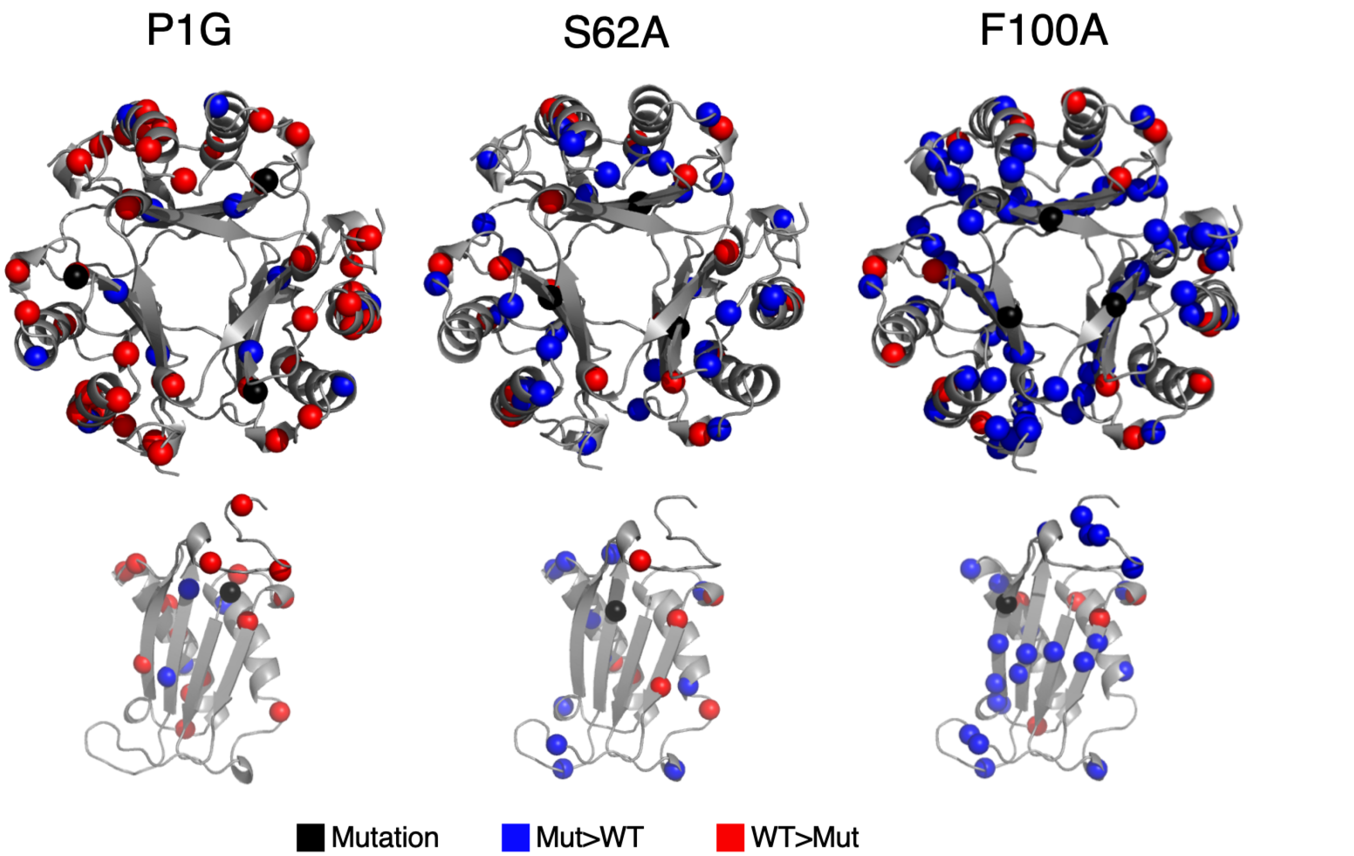

Figure S11. Summary of differences in R_1 and R_2 NMR relaxation parameters caused by allosteric mutations in MIF-2. Residues with R_1R_2 correlations outside the 1.5σ significance cutoff in **Figure 6A** are mapped onto the MIF-2 trimer (top) and monomer (bottom) structures as a difference calculated as $(R_1R_2)_{MUT} - (R_1R_2)_{WT}$. Sites outside of linear correlation where the mutant R_1R_2 is elevated are indicated by blue spheres. Sites outside of linear correlation where the mutant R_1R_2 is depressed are indicated by red spheres. Sites of mutation are indicated by black spheres.

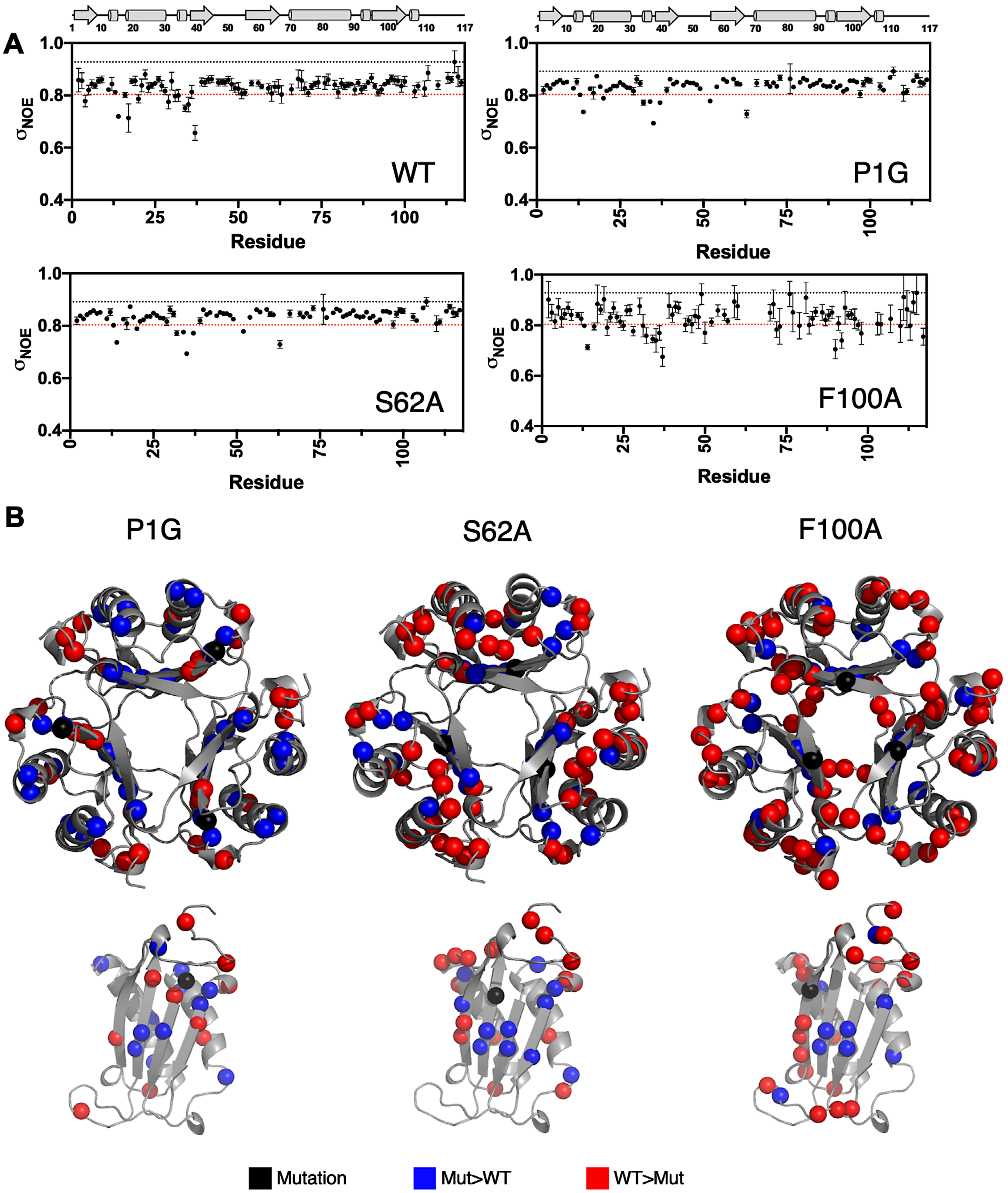

Figure S12. Summary of NMR ^{1}H-[^{15}] NOE relaxation parameters. **(A)** The per-residue ^{1}H-[^{15}] NOE is plotted for wt-MIF-2 and variants P1G, S62A, and F100A. Red dashed lines indicate 1.5σ from the 10% trimmed mean of all NOEs. Dynamic profiles are similar, though S62A and F100A MIF-2 show the most deviation from wt-MIF2. **(B)** Summary of differences in ^{1}H-[^{15}] NOE caused by mutations in MIF-2. Residues outside the 1.5σ significance cutoff in **(A)** are mapped onto the MIF-2 trimer (top) and monomer (bottom) structures as a difference calculated as (^{1}H-[^{15}] NOE)$_{MUT}$ – (^{1}H-[^{15}] NOE)$_{WT}$. Sites where the mutant ^{1}H-[^{15}] NOE is greater are indicated by blue spheres. Sites outside where the mutant ^{1}H-[^{15}] NOE is depressed are indicated by red spheres. Sites of mutation are indicated by black spheres. Cartoons at top show the secondary structure elements of MIF-2 (not to scale).

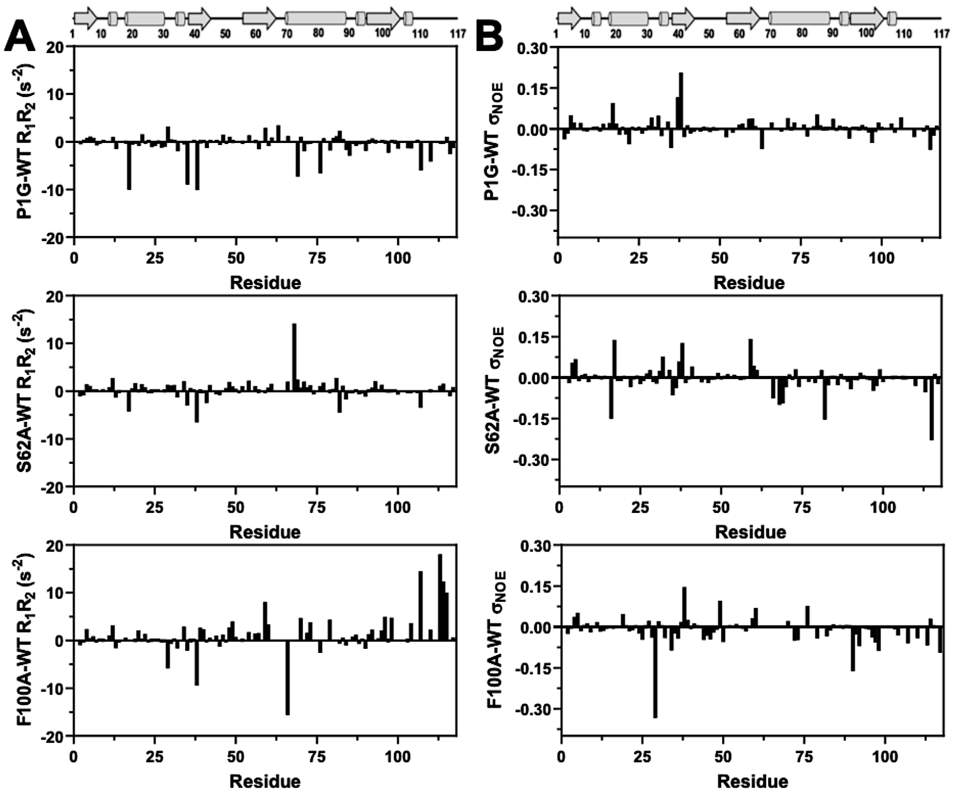

Figure S13. Per-residue differences in R_1R_2 and ^{1}H-[^{15}] NOE relaxation parameters for MIF-2 variants. **(A)** The per-residue R_1R_2 is plotted for P1G, S62A, and F100A as $(R_1R_2)_{MUT}$ – $(R_1R_2)_{WT}$. Positive values indicated a greater dynamic contribution of the mutant while negative values indicated a greater dynamic contribution of wt-MIF2. **(B)** The per-residue ^{1}H-[^{15}] NOE is plotted for P1G, S62A, and F100A as (^{1}H-[^{15}] NOE)$_{MUT}$ – (^{1}H-[^{15}] NOE)$_{WT}$. Positive values indicated a greater dynamic contribution of the mutant while negative values indicated a greater dynamic contribution of wt-MIF2. Cartoons at top show the secondary structure elements of MIF-2 (not to scale).

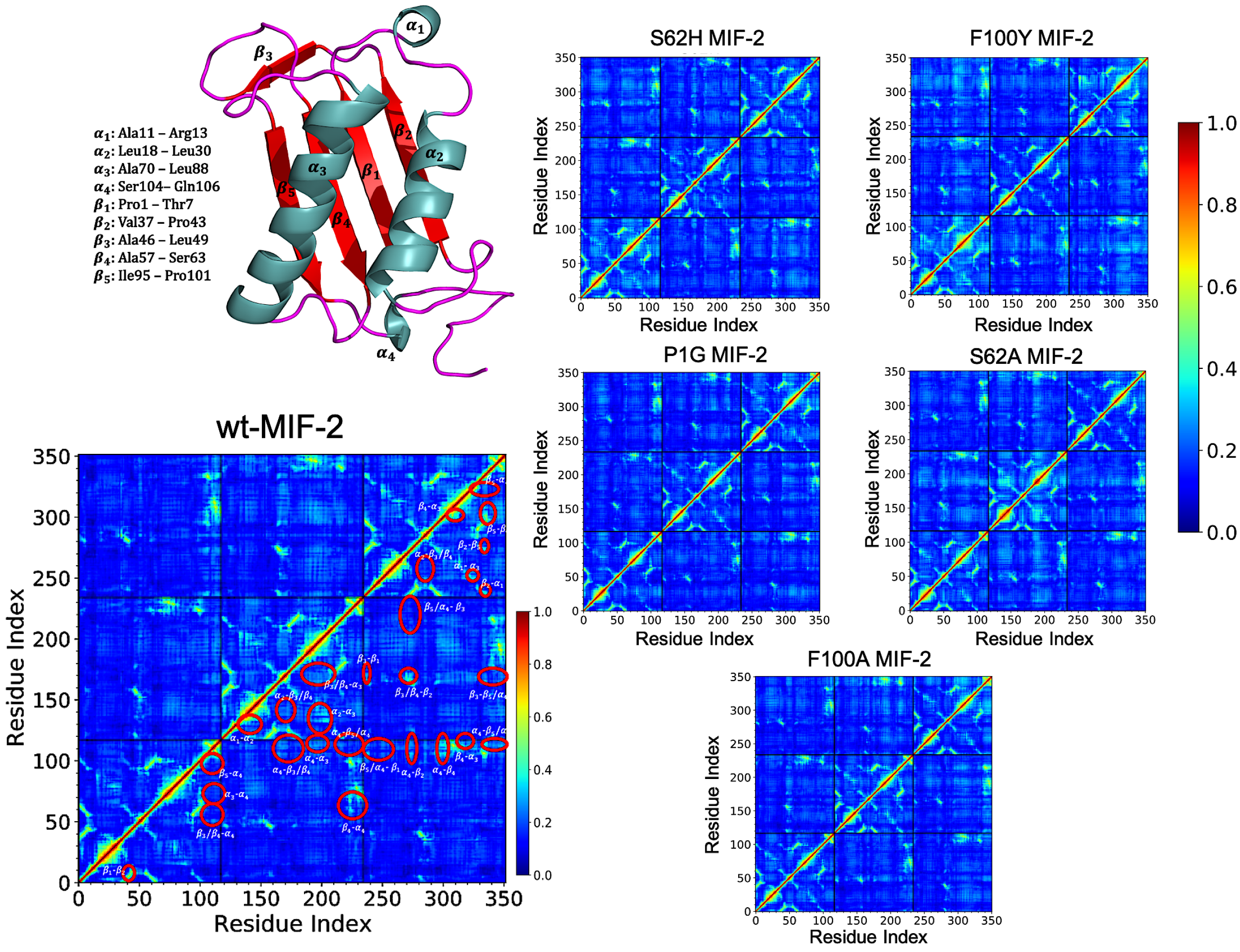

Figure S14. Heat maps for wt-MIF-2 and its S62H, F100Y, P1G, S62A, and F100A variants examining intra-residue correlation during 200 ns MD simulations quantifying RMSD values from alpha carbons of aligned trajectories. Critical secondary structure elements are shown on the MIF monomer and circled in the wt-MIF-2 correlation maps. Each square within the map represents one of the MIF monomers (*i.e.* A, B, or C; bottom-to-top, and left-to-right). The diagonals show the A → A, B → B, and C → C correlations. Red indicates strong correlation between structural elements, while blue indicates little-to-no correlation. In general, S62H and F100Y mutations (mimicking allosteric residues in MIF) increase correlations across the protein, while P1G, S62A and F100A mutations decrease correlations across the protein.

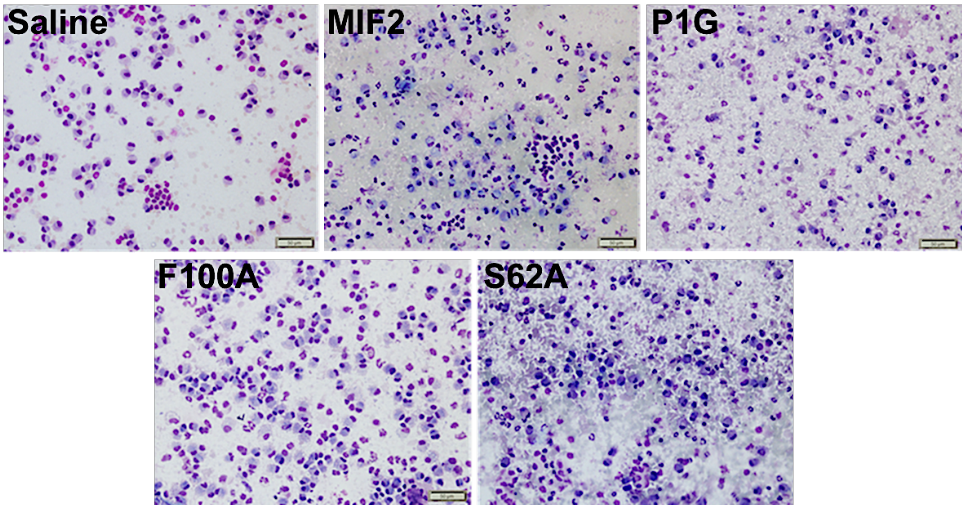

Figure S15. Representative images of Hema staining in BAL fluid cell pellets show decreased neutrophil influx by MIF-2 variants P1G and F100A, compared to wt-MIF-2, administered as a one-time intra-tracheal dose in mice. (n=4 in each group). BAL: bronchoalveolar lavage. The scale bar in these images is 50 μm.
